# Paternal and Maternal Problem Drinking and Lifetime Problem Drinking of Their Adult Children

**DOI:** 10.1017/thg.2023.12

**Published:** 2023-04-24

**Authors:** Pyry N. Sipilä, Anna Keski-Rahkonen, Joni V. Lindbohm, Richard J. Rose, Jaakko Kaprio

**Affiliations:** 1 Clinicum, Department of Public Health, University of Helsinki, Helsinki, Finland; 2 Department of Epidemiology and Public Health, University College London, London, United Kingdom; 3 Department of Psychological and Brain Sciences, Indiana University, Bloomington, Indiana, USA; 4 Institute for Molecular Medicine Finland, University of Helsinki, Helsinki, Finland

**Keywords:** Adult children, alcohol drinking, cohort studies, problem drinking, parents

## Abstract

Parents’ alcohol use is associated with alcohol use of their adolescent offspring, but does this association extend to the adulthood of the offspring? We examined associations of paternal and maternal problem drinking with lifetime problem drinking of their adult offspring prospectively assessed in a population-based Finnish twin-family cohort (FinnTwin16). Problem drinking (Malmö-modified Michigan Alcoholism Screening Test) was self-reported separately by mothers and fathers when their children were 16. The children reported on an extended lifetime version of the same measure during their mid-twenties (21–28 years) and mid-thirties (31–37 years). 1235 sons and 1461 daughters in mid-twenties and 991 sons and 1278 daughters in mid-thirties had complete data. Correlations between fathers’ and their adult children’s problem drinking ranged from .12 to .18. For mothers and their adult children, these correlations ranged from .09 to .14. In multivariate models, adjustment for potential confounders had little effect on the observed associations. In this study, parental problem drinking was modestly associated with lifetime problem drinking of their adult children. This association could be detected even when the children had reached the fourth decade of life.

A number of classic studies have shown that the risk of alcoholism runs in families (Anda et al., 2002; Cotton, 1979; Schuckit & Smith, 1996; Sher et al., 1991). More recent studies have corroborated these findings and extended them to a range of patterns of drinking, including drinking and intoxication frequency and Alcohol Use Disorders Identification Test (AUDIT) scores (Boden et al., 2019; Kendler, Ji et al., 2015; Mahedy et al., 2018; McGovern et al., 2023; Rossow, Felix et al., 2016; Rossow, Keating et al., 2016;). However, the existing studies on the association of parental alcohol use with alcohol use of their children focus on adolescence and the early twenties, and whether these associations extend to later adulthood is uncertain (Mahedy et al., 2018; Rossow, Felix et al., 2016; Rossow, Keating et al., 2016).

A previous study from the United States found that those who were on a high drinking trajectory from age 15 to 25 also had mothers and fathers who on average had a higher drinking frequency, with standardized difference in parental drinking frequency ranging from 0.28 (95% CI [0.02, 0.54]) to 0.55 (95% CI [0.26, 0.84]) (White et al., 2000). Another study from the United States found that maternal drinking frequency was associated with heavy drinking of their 26-year old sons (odds ratio per unit increase in maternal drinking 1.75; 95% CI [1.11, 2.70]) (Englund et al., 2008). Cohort studies from the Nordic countries support these findings. Parental alcohol consumption and binge drinking were associated with alcohol consumption and binge drinking in their children at age 28 (adjusted beta 0.09 [*p* < .001] for alcohol consumption and 0.13 [*p* < .001] for binge drinking; Pedersen & von Soest, 2013). Frequent paternal drinking was also associated with an increased risk of alcohol-related hospitalizations (adjusted hazard ratio 1.73; 95% CI [1.47, 2.04]) and causes of death (adjusted hazard ratio 2.05; 95% CI [1.34, 3.13]) among their sons (Hemmingsson et al., 2017; Landberg et al., 2018). By contrast, in the Northern Finland Birth Cohort 1986, paternal and maternal drinking frequency had only weak correlations with offspring alcohol use disorder at age 28 (point biserial correlations .02–.05 of which only some were statistically significant; Parra et al., 2020). These studies have, however, assessed either alcohol consumption and binge drinking or alcohol-related diagnoses instead of a broader spectrum of alcohol-related problems. Yet, problem drinking affects many people, of which only a fraction receive an alcohol-related diagnosis (Connor et al., 2016; Grant et al., 2015; Mitchell et al., 2012; Nyström et al., 1993).

To our knowledge, only two studies have assessed parental drinking in relation to offspring problem drinking in adulthood, and both had important limitations. In a Finnish study, parental drinking was associated with problem drinking of their 42-year-old sons (Pearson’s *r* .31 [*p* < .001]; Pitkänen et al., 2008), but that study used a measure of problem drinking that had not been validated, and data on parental drinking were partly retrieved from offspring reports. A study from the United States found that heavy parental drinking was associated with symptoms of alcohol abuse and dependence among their offspring at mid-thirties (Pearson’s *r* .12–.16; *p* < .001]; Merline et al., 2008), but this was based on attributions of parental drinking by the offspring, not parental self-assessments.

Many confounding factors may also be present. In their systematic review, Rossow, Keating et al. (2016) proposed factors that could induce spurious associations between parental and offspring alcohol use. These included shared local environment, cultural and religious factors, and parental comorbidities and temperament. Related characteristics have also been associated with drinking behavior in earlier studies, supporting the idea that they might potentially confound the association between parental and offspring problem drinking (Edlund et al., 2010; Gauffin et al., 2013; Kestilä et al., 2008; Winter et al., 2002). For example, local environment, availability of alcoholic beverages, and cultural and religious attitudes could influence the drinking of both parents and offspring, consequently creating associations between these two that are not related to parental drinking per se. Parental temperament could influence parental drinking and, through inheritance, also the temperament and drinking of the offspring.

To address limitations of earlier studies, we examined how paternal and maternal problem drinking are associated with lifetime problem drinking of their adult offspring, drawing on data from a population-based cohort study of Finnish twins and their parents. Problem drinking was prospectively individually reported by the different informants (parents and offspring) using comparable and validated measures. The offspring were assessed in their mid-twenties and mid-thirties, when they were approaching the age at which problem drinking of their parents was recorded, a time when most offspring had been living away from their childhood home for well over a decade. Our focus was on overall associations and not on potential mediating factors. However, we considered the confounding effects of several proposed confounders and the mediating effects of problem drinking in mid-twenties.

## Materials and Methods

### Study Design and the Study Cohort

The population-based FinnTwin16 cohort identified all twin births in Finland across five consecutive years (1975–1979) from the Finnish Population Information System (Kaidesoja et al., 2019). At baseline (wave 1), questionnaires were sent 10 times per year over 60 months to parents and twins such that the twins from the five successive birth cohorts received their questionnaires as close to their 16th birthday as possible. Follow-up waves were conducted at age 17 (wave 2), 18.5 (wave 3), 21–28 (wave 4, mid-twenties), and 31–37 (wave 5, mid-thirties).

In wave 1, 89.9% of the invited individual twins replied. Of those who had replied in wave 1, 85.4% replied also in wave 4 and 66.4% in both waves 4 and 5. In total, 5240 individuals (2415 men and 2825 women) returned a mailed questionnaire in wave 4 (mean age 24.5, range 21–28) and 4409 (1963 men and 2446 women) an electronic questionnaire in wave 5 (mean age 34.1, range 31–37) (Supplementary Figure 1). The main analyses of the present paper comprise lifetime drinking participants who had full data on relevant covariates, a total of 2696 participants (1235 men and 1461 women) in wave 4 and 2269 participants (991 men and 1278 women) in wave 5. Supplementary Tables 1 and 2 present an attrition analysis of study variables by inclusion versus exclusion to the study sample. Information on fathers’ and mothers’ problem drinking and potential confounders was obtained with a mailed questionnaire administered at baseline, when the sons and daughters were aged 16 years.

The Ethics Committee of the Department of Public Health, University of Helsinki and the Institutional Review Board of Indiana University approved the data collection (waves 1–3) and analysis. Data collection for waves 4 and 5 was approved by the Ethics Committees of the Hospital Districts of Helsinki and Uusimaa and the Hospital District of Central Finland respectively.

### Problem Drinking

We measured parental problem drinking with the Malmö-modified Michigan Alcoholism Screening Test (Mm-MAST), which was designed for Nordic cultures. It consists of nine yes–no questions yielding scores ranging from 0 to 9 (Supplementary Table 3) (Kristenson & Trell, 1982). High scores are a self-appraisal of drinking-related problems, and they are positively correlated with total alcohol consumption, intoxication frequency, and heavy drinking (Nyström et al., 1993; Seppä et al., 1992; Seppä et al., 1990). Fathers and mothers self-reported on their problem drinking in wave 1 when their sons and daughters were age 16. Because both fathers’ and mothers’ Mm-MAST scores had highly skewed distributions, we categorized them by collapsing high scores into a single category (≥4). Suggested cut-offs to identify problem drinking vary from 2 to 4 (Kristenson & Trell, 1982; Nyström et al., 1993; Rose et al., 1999; Seppä et al., 1992; Seppä et al., 1990). Accordingly, we analysed both fathers’ and mothers’ Mm-MAST in five categories comprising those who scored 0, 1, 2, 3, and ≥4.

The sons and daughters self-reported on their problem drinking in their mid-twenties (wave 4, age 21–28 years) and again in their mid-thirties (wave 5, age 31–37) with an extended, 11-item, lifetime version of Mm-MAST (Mm-MAST-11, Supplementary Table 3). The two additional items were added to make the scale a more sensitive measure of alcohol abuse and dependence (Kaprio et al., 2002), and to increase its internal consistency (in our sample, Cronbach’s alpha was .69 for fathers and .66 for mothers, for whom the original scale was used, but .78 and .75 for sons and daughters at mid-twenties and .78 and .77 for sons and daughters at mid-thirties, for whom the amended scale was used).

For both measures of problem drinking, Mm-MAST and Mm-MAST-11, we included all responses with no more than two missing items and substituted for the missing items the mean score of the available items of each included respondent. Substitutions were made for 6% of fathers and mothers and 1% of their adult children. Further, respondent parents (but not offspring) were instructed to skip the entire scale if they did not drink at all. Therefore, they received an Mm-MAST score of zero if all items were missing and they reported no alcohol drinks during the past year.

### Heavy Drinking Occasions

We complemented our analyses of problem drinking with analyses of current heavy drinking occasions because they are an indicator of alcohol use disorder (Rehm et al., 2017), mortality (Sipilä et al., 2016), and high total alcohol consumption (Gmel et al., 2011). We defined heavy drinking occasions as consuming ‘within one occasion … more than five bottles of beer, or more than a bottle of wine, or more than half a bottle of hard liquor (or a corresponding amount of alcohol)’, which corresponds to consuming more than five standard drinks (>60 g of pure alcohol) on a single occasion. Fathers and mothers self-reported on their heavy drinking occasions in wave 1. We analysed their reports in five categories: (1) never, (2) once a year or less often, (3) a few times a year, (4) about once a month, and (5) about once a week or more often. From questionnaires administered to sons and daughters, we recorded current heavy drinking occasions at mid-twenties and mid-thirties in 10 categories, converting them to a continuous measure of heavy drinking occasions per year (range 0–365). The 10 categories and the corresponding numbers of yearly occasions were: I don’t use alcohol at all (0), never (0), once a year or less often (0.75), 3–4 times a year (3.5), about once in two months (6), about once a month (12), a couple of times a month (24), about once a week (52), about twice a week (104) and daily (365). As with problem drinking, lifetime abstainers were excluded.

### Lifetime Abstinence

Because the determinants of abstinence are known to be different from the determinants of drinking habits among those who drink (Maes et al., 1999; Rose et al., 2001; Rose et al., 1999; Viken et al., 2007), we excluded from our primary analyses those who reported themselves to be lifetime abstainers. Consequently, we excluded fathers of 317 adult children (7%), mothers of 617 adult children (12%), 215 adult children in their mid-twenties (4%), and 118 adult children in their mid-thirties (3%). To test the effects of these exclusions, we conducted sensitivity analyses with abstainers included.

### Potential Confounders

We stratified all models by sex. Partial adjustment for age was inherent to data collection, which was done in waves at defined ages. As detailed below, we also adjusted the multivariate models for best available proxies for socioeconomic status of the family, family situation, and parental and family characteristics suggested to be potentially important in a systematic review by Rossow, Keating et al. (2016). All covariates were measured in wave 1 when the twins were 16 years old. To avoid over-adjustment, we did not adjust for offspring characteristics, because they may mediate the associations between parental and offspring problem drinking.

Fathers and mothers self-reported their education in wave 1. This information was analysed as a dichotomy separately for fathers and mothers: academic (completed high school) vs. nonacademic (did not attend or complete high school).

Family structure was self-reported by the sons and daughters in adolescence (wave 1) and analysed as a dichotomy of whether (or not) they were living with both biological parents at age 16.

Area of residence reflected the local environment and cultural milieu. It was retrieved from the Finnish Population Information System in the last year of wave 1 and was classified according to the European Union’s Nomenclature of Territorial Units (Rose et al., 1999; Statistics Finland, 1998). We analysed area of residence in three categories characterized by high, low, and intermediate average alcohol consumption (capital area, Mid-Finland and West coast, and the rest of Finland respectively; Simpura & Lahti, 1988).

We measured fathers’ and mothers’ religiosity using the religious fundamentalism content scale of the Minnesota Multiphasic Personality Inventory (MMPI) consisting of 12 yes–no items about religious practice and beliefs, with an emphasis on the tenets of Christianity, which is the majority religion in Finland (Sipilä et al., 2017; Wiggins, 1966; Winter et al., 2002). The observed scores spanned the entire theoretical range from 0 to 12, with higher scores indicating higher religiosity. The scale was included in the parents’ questionnaire mailed to them at baseline (wave 1). We included those answering at least nine items, with the mean score of the available items substituted for missing items.

We assessed a risk-relevant dimension of fathers’ and mothers’ personality using a 50-item social deviance scale (the Pd or ‘Psychopathic deviate’ scale of the MMPI), a proxy for parental comorbidity and temperament. Although the theoretical range is from 0 to 50, the observed scores ranged from 5 to 42 for fathers and from 3 to 36 for mothers with higher scores reflecting higher social deviance. The scale is also associated with problem drinking (Mustanski et al., 2003; Viken et al., 2007) and captures part of the genetic risk for externalizing disorders in the family, as common genetic background contributes to the comorbidity of externalizing disorders (Kendler et al., 2003). The scale was included in the parents’ questionnaire at baseline (wave 1). We included participants who had completed at least 40 items. Mean score of the available items was substituted for missing items. The proportion of respondents with data available for problem drinking, religiosity, and the Pd scale before and after the substitutions are reported in Supplementary Methods.

### Statistical Analysis

We estimated polychoric correlations between ordinal variables (Kolevnikov & Angeles, 2004), also treating sons’ and daughters’ reports on heavy drinking occasions as ordinal for the purpose of correlation analysis. We assessed the underlying bivariate normality assumption with Pearson’s chi-squared tests; significant *p* values warrant careful interpretation of the estimated polychoric correlations. For that reason, we also report Spearman’s rank correlation coefficients (rho) for comparison for correlations with evidence for violation of the underlying bivariate normality assumption. Because sons’ and daughters’ problem drinking was assessed continuously, we estimated polyserial correlations between them and ordinal variables (Kolevnikov & Angeles, 2004).

In multivariate analysis, we estimated sons’ and daughters’ problem drinking (mean Mm-MAST-11) using multiple linear regression analysis. In contrast, we used generalized linear models with log link and Poisson distribution to estimate sons’ and daughters’ mean heavy drinking occasions per year because the distribution of heavy drinking occasions was highly skewed. Further, we applied robust variance estimators to get unbiased confidence intervals despite heteroscedasticity and overdispersion (Cameron & Trivedi, 2010; Wooldridge, 2010). To allow for nonlinear relations, and thus, to reduce residual confounding, we modelled religiosity and personality with restricted cubic splines with three knots at 10th, 50th, and 90th percentiles (Harrell, 2015). We tested for differences in associations of sons’ versus daughters’ problem drinking with fathers’ versus mothers’ problem drinking using Wald tests. We adjusted all confidence intervals for clustering within twin pairs (Williams, 2000). All *p* values are two-sided; *p* < .05 was considered statistically significant. We analysed the data using Stata statistical software (StataCorp, College Station, TX, USA).

## Results

### Descriptive Statistics

Table 1 summarizes problem drinking, heavy drinking occasions, and potential confounders among study participants who were not lifetime abstainers. Fathers reported more problem drinking than mothers: 20.9% of fathers (95% CI [18.8, 23.1]), but only 8.7% of mothers (95% CI [7.4, 10.3]) scored ≥4 on the Mm-MAST. Similarly, sons reported more problem drinking than did daughters (e.g., mean lifetime Mm-MAST-11 at mid-thirties was 4.5 for sons and 3.0 for daughters, *p* for difference <.001).

### Correlations

Table 2 presents polyserial and polychoric correlations between measures of problem drinking and heavy drinking occasions. The correlations of these measures of drinking with covariates are shown in Supplementary Table 4. Correlations between parents’ problem drinking and their children’s lifetime problem drinking were very modest (.09–.18). Correlations between parents’ and their children’s current heavy drinking occasions were similar (.12–.19). Correlation between fathers’ and mothers’ problem drinking was .40 and between fathers’ and mothers’ heavy drinking occasions it was .46. The correlations remained similar when the analyses were restricted to those who were living with both biological parents at age 16 (Supplementary Table 5).

### Multivariate Models of Problem Drinking

We included fathers’ and mothers’ problem drinking simultaneously in the same model to assess their associations with offspring’s problem drinking independently from each other (Tables 3 and 4). Fathers’ high problem drinking was associated with higher lifetime problem drinking in their sons and daughters measured in their mid-twenties and mid-thirties, even after adjustment for mothers’ problem drinking. *P* values for linear trend were significant for all these comparisons. The associations of mothers’ problem drinking with lifetime problem drinking in offspring at both mid-twenties and mid-thirties showed similar patterns to fathers’ problem drinking, although the associations failed to reach statistical significance for many comparisons. *P* values for linear trend were significant for the associations of maternal problem drinking with sons’ lifetime problem drinking, but not with daughters’ lifetime problem drinking. Yet, the differences between the associations of paternal and maternal problem drinking with offspring lifetime problem drinking were not statistically significant and, as indicated by the highly overlapping confidence intervals, the associations of fathers’ and mothers’ problem drinking with their sons’ versus daughters’ lifetime problem drinking were of comparable strength.

We tested whether adjustments for area of residence, family structure, and fathers’ and mothers’ education, religiosity and personality would affect our results. The magnitude and direction of the associations remained the same (Model 1 vs. Model 2 in Tables 3 and 4). Further, the observed associations were slightly stronger when lifetime abstainers were included, suggesting that our approach of excluding lifetime abstainers was conservative (Supplementary Tables 6–8).

### Mediating Role of Problem Drinking at Mid-Twenties

The association of fathers’ problem drinking with lifetime problem drinking of their offspring at mid-thirties was considerably attenuated when adjusted for lifetime problem drinking of their offspring at mid-twenties (Table 5). The associations of mothers’ problem drinking were attenuated even more.

### Multivariate Models of Heavy Drinking Occasions

In multivariate analyses, the associations between fathers’ and mothers’ and their adult children’s heavy drinking occasions were often statistically nonsignificant (Supplementary Figures 2–5).

## Discussion

In this population-based cohort study, we observed a modest association between paternal problem drinking and lifetime problem drinking of their adult children. This association could be detected even when the children’s problem drinking was assessed at mid-thirties(i.e., almost two decades after the fathers had reported their problem drinking). And this association remained similar after controlling for family and individual parental characteristics. Maternal problem drinking showed a similar, but less statistically robust association with problem drinking of adult offspring.

Many studies have found associations between alcohol use of parents and their children in adolescence and in their twenties (Mahedy et al., 2018; Rossow, Felix et al., 2016; Rossow, Keating et al., 2016; Yap et al., 2017). This study extends these findings into lifetime problem drinking measured during the third and fourth decades of life (mean age 24.5 and 34.1 years, respectively), when offspring have been living in adulthood for well over a decade and are approaching the age at which their parents’ problem drinking was assessed (fathers’ mean age 46.0 years, mothers’ mean age 44.0 years). However, the associations of parental problem drinking with offspring lifetime problem drinking at mid-thirties could be substantially explained by offspring lifetime problem drinking at mid-twenties, highlighting the importance of the third decade of life as a critical period for problem drinking.

Our results are compatible with the few earlier studies on the association between parental alcohol use and later alcohol use among adult offspring. In a cohort study from Norway, the correlations of combined parental alcohol consumption and binge drinking with alcohol consumption and binge drinking of their children ranged from .09 to .16 (Pedersen & von Soest, 2013). In our study, correlations ranged from .09 to .18. In other cohort studies from the United States, both mothers’ and fathers’ drinking frequency were associated with a higher probability of being on a heavy drinking trajectory from late adolescence to mid-twenties (White et al., 2000), and parental heavy drinking was associated with heavy drinking and symptoms of alcohol abuse and dependence of offspring at age 35 years (Pearson’s *r* .12–.16; Merline et al., 2008). In a Finnish cohort study, parental drinking was associated with problem drinking among their sons in their forties (Pearson’s *r* 0.31; Pitkänen et al., 2008).

The correlations of .09–.18 observed in our study indicate that parental problem drinking might explain 1–3% of the variation in offspring problem drinking. This is relatively modest, but well consistent with previous estimates from observational (1–10%) and genetic studies (single-nucleotide polymorphism [SNP] heritability 4%; Liu et al., 2019; Merline et al., 2008; Pedersen & von Soest, 2013; Pitkänen et al., 2008). These findings suggest that there is substantial variation in problem drinking that cannot be traced back to the problem drinking of previous generations. Consequently, although potentially very beneficial for the targeted individuals, interventions addressing problem drinking on the individual level can be expected to have only modest effects on the problem drinking of subsequent generations. This highlights the need for multigenerational approaches that pay adequate attention to the situation and needs of each individual generation.

In our study, maternal problem drinking showed weaker correlations with lifetime problem drinking of adult offspring than did paternal problem drinking. When both paternal and maternal problem drinking were simultaneously considered in the same model, maternal problem drinking had again weaker associations with offspring problem drinking than did paternal problem drinking. Although the difference was not statistically significant, it was consistent across several analyses. However, in earlier studies the findings have been inconsistent (Alati et al., 2014; Mahedy et al., 2018; Mares et al., 2011; White et al., 2000). Further studies are necessary to examine the differential associations of fathers’ versus mothers’ drinking with their offspring’s drinking.

Associations between parental and offspring alcohol use are at least partly explained by dispositional genetic factors inherited by the children (Hopfer et al., 2003; Liu et al., 2019; Verhulst et al., 2015; Walters et al., 2018). Those could be further assessed using genetic data and co-twin designs. However, at least the familial aggregation of alcohol use disorder cannot be fully explained by shared genetic predisposition (Kendler, Ji et al., 2015; Kendler, Ohlsson et al., 2015). Besides shared genetic dispositions, assortative mating can substantially contribute to parent–offspring similarities (Ruby et al., 2018). Consistent with significant assortative mating, paternal and maternal problem drinking were correlated at .40 in our data, although this correlation may also reflect changes in the drinking patterns of one or both of the parents during the time they have lived together.

Nongenetic familial mechanisms may also contribute to the associations between parental and offspring problem drinking. Parental problem drinking may influence parenting and increase stress in the family (Leonard & Eiden, 2007), possibly activating a genetic predisposition to alcohol use in offspring (Jacob et al., 2003; Rossow, Keating et al., 2016). Parental monitoring, discipline, and alcohol rules may mediate these effects (Latendresse et al., 2008; Sharmin et al., 2017). In addition, parents who drink may have more approving attitudes towards alcohol drinking, will probably have alcohol in the household, and may even be more inclined to supply alcohol to their children, which may increase the children’s problem drinking (Maggs & Staff, 2018; Mattick et al., 2018; Yap et al., 2017). Further, family structure (which we controlled for) and social networks may be both confounders and mediators (Delucchi et al., 2008; Leonard & Rothbard, 1999). However, these proposed nongenetic familial mechanisms mainly operate when the parents and their children live together. Future studies are needed to assess whether their effects extend to the adulthood of the offspring.

This study had some limitations. Our measures were based on self-reports (with the exception of age, sex, and area of residence, which were retrieved from the Finnish Population Information System) and are subject to reporting error. Childhood characteristics were not assessed, because baseline assessments were in mid-adolescence. Data on offspring problem drinking using Mm-MAST were not collected at baseline at age 16, but the duration and quantity of drinking will have been quite limited for most children at that age. The measures of problem drinking administered to parents and offspring were not identical. The sons’ and daughters’ questionnaires assessed lifetime problem drinking and could not differentiate between current and past drinking problems. Nonetheless, when assessing current heavy drinking occasions reported by the sons and daughters in their mid-twenties and mid-thirties, we found similar correlations.

As with most longstanding studies, attrition and missing information on individual items was considerable and may cause bias; both abstainers and heavy drinkers may have been more prone to nonresponse (Dawson et al., 2014). Heavy-drinking individuals may underreport their drinking (Northcote & Livingston, 2011), and drinking may vary over time (Knott et al., 2018). Further, parental questionnaires did not specifically enquire about past drinking problems, which may have led to misclassification of former problem drinkers and underestimation of true associations. Moreover, the determinants of problem drinking are complex (Stone et al., 2012). Some residual confounding due to unidentified or imprecisely measured covariates is likely present. Finally, lack of statistical significance in some comparisons cannot be interpreted as evidence for no association, as our confidence intervals are also compatible with modest associations.

The main strength of this study is its intergenerational design, with participants of each generation themselves reporting their alcohol use. We investigated a prospective population-based cohort with high response rates. We assessed both problem drinking and heavy drinking occasions across time with validated measures. In addition, both of the offspring assessments were completed 10 years apart in adulthood when patterns of alcohol use begin to stabilize (Knott et al., 2018; Maggs & Schulenberg, 2004).

In conclusion, in this population-based cohort study, parental problem drinking was modestly associated with lifetime problem drinking of their adult children. This association could be detected even when the children had reached the fourth decade of life.

## Supplementary Material

Supplementary Material

## Figures and Tables

**Table 1.** Basic characteristics of lifetime drinkers in the study cohort

	Sons		Daughters		*p* value for difference between sons and daughters
	*N*	Mean (*SD*)	*N*	Mean (*SD*)	
Lifetime problem drinking (Mm-MAST-11) at mid-twenties	1235	4.7 (2.8)	1461	3.5 (2.4)	<.001
Lifetime problem drinking (Mm-MAST-11) at mid-thirties	991	4.5 (2.8)	1278	3.0 (2.5)	<.001
Heavy drinking occasions (per year) at mid-twenties	1280	29.6 (31.8)	1497	14.5 (20.5)	<.001
Heavy drinking occasions (per year) at mid-thirties	1028	24.1 (33.9)	1317	7.9 (18.5)	<.001
Fathers’ religiosity	1235	3.4 (2.8)	1461	3.3 (2.7)	.40
Mothers’ religiosity	1235	4.6 (2.7)	1461	4.6 (2.7)	.95
Fathers’ personality (Pd scale)	1235	15.8 (4.9)	1461	16.1 (5.1)	.16
Mothers’ personality (Pd scale)	1235	14.8 (4.8)	1461	15.1 (4.9)	.15
	Sons		Daughters		
	*N*	%	*N*	%	
**Fathers’ problem drinking (Mm-MAST)**	1235		1461		.59
0	322	26.1	391	26.8	
1	243	19.7	294	20.1	
2	218	17.7	276	18.9	
3	195	15.8	194	13.3	
≥4	257	20.8	306	20.9	
**Mothers’ problem drinking (Mm-MAST)**	1235		1461		.64
0	572	46.3	649	44.4	
1	285	23.1	327	22.4	
2	180	14.6	222	15.2	
3	92	7.4	134	9.2	
≥4	106	8.6	129	8.8	
**Fathers’ heavy drinking occasions**	1280		1497		.37
- never	206	16.1	265	17.7	
- once a year or less often	180	14.1	173	11.6	
- a few times a year	387	30.2	465	31.1	
- about once a month	246	19.2	270	18.0	
- about once a week or more often	261	20.4	324	21.6	
**Mothers’ heavy drinking occasions**	1280		1497		1.00
- never	640	50.0	732	48.9	
- once a year or less often	212	16.6	256	17.1	
- a few times a year	255	19.9	304	20.3	
- about once a month	112	8.8	124	8.3	
- about once a week or more often	61	4.8	81	5.4	
**Fathers’ education**	1235		1461		.60
- academic	287	23.2	325	22.2	
- nonacademic	948	76.8	1136	77.8	
**Mothers’ education**	1235		1461		.85
- academic	357	28.9	428	29.3	
- nonacademic	878	71.1	1033	70.7	
**Area of residence in adolescence**	1235		1461		.88
- capital area	312	25.3	356	24.4	
- Mid-Finland or West coast	175	14.2	206	14.1	
- rest of Finland	748	60.6	899	61.5	
**Living with both parents in adolescence**	1235		1461		.04
- yes	1116	90.4	1278	87.5	
- no	119	9.6	183	12.5	

Note: The numbers represent those without missing information on the characteristic in question. CI, confidence interval; *SD*, standard deviation; Mm-MAST, Malmö-modified Michigan Alcoholism Screening Test (original 9-item version); Mm-MAST-11, Malmö-modified Michigan Alcoholism Screening Test (extended 11-item version); Pd scale, Pd or “Psychopatic deviate” scale of the Minnesota Multiphasic Personality Inventory.

**Table 2.** Correlations between measures of problem drinking and heavy drinking occasions

Polyserial correlations	Sons’ lifetime Mm-MAST-11 at mid-twenties (*n* = 1235)	Daughters’ lifetime Mm-MAST-11 at mid-twenties (*n* = 1461)	Sons’ lifetime Mm-MAST-11 at mid-thirties (*n* = 991)	Daughters’ lifetime Mm-MAST-11 at mid-thirties (*n* = 1278)
Fathers’ Mm-Mast	0.18**	0.12**	0.16**	0.18**
Mothers’ Mm-Mast	0.12**	0.09*	0.14**	0.11**
Polychoric correlations				
	Sons’ heavy drinking occasions at mid-twenties (*n* = 1280)	Daughters’ heavy drinking occasions at mid-twenties (*n* = 1497)	Sons’ heavy drinking occasions at mid-thirties (*n* = 1028)	Daughters’ heavy drinking occasions at mid-thirties (*n* = 1317)
Fathers’ heavy drinking occasions	019**^a^ (rho = 0.18**)	0.13**^a^ (rho = 0.12**)	0.13**	0.13**
Mothers’ heavy drinking occasions	0.16**^a^ (rho = 0.13**)	0.17**	0.15**^a^ (rho = 0.13**)	0.12**
	Fathers’ Mm-Mast (*n* = 1511)	Mothers’ Mm-Mast (*n* = 1511)	Fathers’ heavy drinking occasions (*n* = 1511)	Mothers’ heavy drinking occasions (*n* = 1511)
Fathers’ Mm-Mast	1	0.40**^a^ (rho = 0.34**)	0.71**	0.32**
Mothers’ Mm-Mast		1	0.36**	0.67**^a^ (rho = 0.55**)
Fathers’ heavy drinking occasions			1	0.46**^a^ (rho = 0.39**)
Mothers’ heavy drinking occasions				1

Note:

* *p* < .01

** *p* < .001.

a Pearson’s chi-squared test indicates violation of the underlying bivariate normality assumption. Spearman’s rank order correlation coefficients (rho) given for comparison in parentheses for those polychoric correlations for which there was evidence for violation of the underlying bivariate normality assumption. Lifetime abstainers were excluded from the analysis.

Mm-MAST, Malmö-modified Michigan Alcoholism Screening Test (original 9-item version); Mm-MAST-11, Malmö-modified Michigan Alcoholism Screening Test (extended 11-item version).

**Table 3.** Association of fathers’ and mothers’ problem drinking with lifetime problem drinking of offspring at mid-twenties

	*N*	Model 1 *(basic model)*		Model 2 *(multiply-adjusted model)*	
		β (95% CI)	*p* value^b^	β (95% CI)	*p* value^b^
**SONS**					
**Fathers’ problem drinking (Mm-MAST)**	1235				
0^a^	322	0		0	
1	243	0.20 (−0.31, 0.71)	.45	0.14 (−0.36, 0.65)	.58
2	218	0.67 (0.13, 1.21)	.015	0.58 (0.04, 1.13)	.035
3	195	0.93 (0.39, 1.47)	.001	0.91 (0.37, 1.45)	.001
≥4	257	0.98 (0.45, 1.51)	<.001	0.85 (0.30, 1.40)	.003
(*p* value for linear trend)			(<.001)		(<.001)
**Mothers’ problem drinking (Mm-MAST)**	1235				
0^a^	572	0		0	
1	285	−0.30 (−0.74, 0.13)	.17	−0.26 (−0.70, 0.18)	.24
2	180	0.19 (−0.32, 0.71)	.46	0.23 (−0.29, 0.74)	.39
3	92	0.53 (−0.14, 1.21)	.12	0.49 (−0.18, 1.16)	.15
≥4	106	0.63 (−0.06, 1.32)	.072	0.58 (−0.13, 1.30)	.11
(*p* value for linear trend)			(.029)		(.046)
**DAUGHTERS**					
**Fathers’ problem drinking (Mm-MAST)**	1461				
0^a^	391	0		0	
1	294	0.29 (−0.14, 0.73)	.18	0.28 (−0.16, 0.71)	.21
2	276	0.19 (−0.22, 0.60)	.37	0.16 (−0.25, 0.58)	.44
3	194	0.56 (0.09, 1.04)	.019	0.52 (0.04, 1.00)	.032
≥4	306	0.62 (0.21, 1.03)	.003	0.53 (0.10, 0.95)	.015
(*p* value for linear trend)			(.002)		(.011)
**Mothers’ problem drinking (Mm-MAST)**	1461				
0^a^	649	0		0	
1	327	0.07 (−0.28, 0.43)	.69	0.07 (−0.28, 0.43)	.69
2	222	0.05 (−0.38, 0.48)	.81	0.07 (−0.37, 0.50)	.76
3	134	0.41 (−0.06, 0.88)	.087	0.41 (−0.06, 0.89)	.088
≥4	129	0.46 (−0.07, 0.98)	.089	0.45 (−0.09, 1.00)	.10
(*p* value for linear trend)			(.051)		(.056)

Note: Model 1 includes simultaneously fathers’ and mothers’ Mm-MAST. Model 2 includes simultaneously fathers’ and mothers’ Mm-MAST + adjustments for fathers’ religiosity, mothers’ religiosity, fathers’ personality, mothers’ personality, fathers’ education, mothers’ education, area ofresidence, and family structure. Lifetime abstainers excluded. Offspring problem drinking measured using Mm-MAST-11.

a Reference category

b *p* value for difference with the reference category.

CI, confidence interval; Mm-MAST, Malmö-modified Michigan Alcoholism Screening Test (original 9-item version); Mm-MAST-11, Malmö-modified Michigan Alcoholism Screening Test (11-item version).

**Table 4.** Association of fathers’ and mothers’ problem drinking with lifetime problem drinking of offspring at mid-thirties

	*N*	Model 1 *(basic model)*		Model 2 *(multiply-adjusted model)*	
		β (95% CI)	*p* value^b^	β (95% CI)	*p* value^b^
**SONS**					
**Fathers’ problem drinking (Mm-MAST)**	991				
0^a^	264	0		0	
1	191	−0.10 (−0.67, 0.46)	.72	−0.20 (−0.77, 0.36)	.48
2	187	0.62 (0.06, 1.18)	.030	0.61 (0.05, 1.17)	.033
3	152	1.11 (0.50, 1.72)	<.001	1.16 (0.55, 1.77)	<.001
≥4	197	0.66 (0.08, 1.24)	.025	0.67 (0.06, 1.28)	.03
(*p* value for linear trend)			(<.001)		(<.001)
**Mothers’ problem drinking (Mm-MAST)**	991				
0^a^	459	0		0	
1	222	0.01 (−0.48, 0.51)	.96	0.06 (−0.44, 0.55)	.82
2	148	0.05 (−0.49, 0.59)	.86	0.06 (−0.48, 0.60)	.83
3	75	0.90 (0.17, 1.62)	.015	0.82 (0.09, 1.56)	.028
≥4	87	0.83 (0.14, 1.52)	.018	0.69 (−0.05, 1.43)	.067
(*p* value for linear trend)			(.007)		(.026)
**DAUGHTERS**					
**Fathers’ problem drinking (Mm-MAST)**	1278				
0^a^	344	0		0	
1	258	0.10 (−0.33, 0.53)	.65	0.10 (−0.34, 0.54)	.65
2	244	0.15 (−0.29, 0.60)	.50	0.11 (−0.33, 0.55)	.62
3	178	0.80 (0.24, 1.36)	.005	0.76 (0.19, 1.34)	.009
≥4	254	1.01 (0.53, 1.48)	<.001	0.93 (0.43, 1.43)	<.001
(*p* value for linear trend)			(<.001)		(<.001)
**Mothers’ problem drinking (Mm-MAST)**	1278				
0^a^	550	0		0	
1	300	−0.04 (−0.43, 0.35)	.84	0.01 (−0.38, 0.41)	.96
2	192	0.10 (−0.39, 0.59)	.69	0.10 (−0.39, 0.60)	.69
3	125	0.25 (−0.32, 0.81)	.39	0.26 (−0.30, 0.82)	.37
≥4	111	0.59 (−0.02, 1.19)	.057	0.54 (−0.06, 1.15)	.076
(*p* value for linear trend)			(.063)		(.080)

Note: Model 1 includes simultaneously fathers’ and mothers’ Mm-MAST. Model 2 includes simultaneously fathers’ and mothers’ Mm-MAST + adjustments for fathers’ religiosity, mothers’ religiosity, fathers’ personality, mothers’ personality, fathers’ education, mothers’ education, area of residence, and family structure. Lifetime abstainers excluded. Offspring problem drinking measured using Mm-MAST-11.

a Reference category

b *p* value for a difference with the reference category.

CI, confidence interval; Mm-MAST, Malmö-modified Michigan Alcoholism Screening Test (original 9-item version); Mm-MAST-11, Malmö-modified Michigan Alcoholism Screening Test (11-item version).

**Table 5.** The contribution of offspring problem drinking at mid-twenties on the association of fathers’ and mothers’ problem drinking with lifetime problem drinking of offspring at mid-thirties

	*N*	Model 2 *(multiply-adjusted model)*		Model 3 *(as model 2 + additionally adjusted for mediation by problem drinking at mid-twenties*	
		β (95% CI)	*p* value^b^	β (95% CI)	*p* value^b^
**SONS**					
**Fathers’ Mm-MAST**	904				
0^a^	241	0		0	
1	174	−0.06 (−0.65, 0.53)	.84	−0.10 (−0.54, 0.33)	.64
2	173	0.70 (0.12, 1.27)	.018	0.42 (0.01, 0.83)	.043
3	137	1.27 (0.65, 1.89)	.000	0.68 (0.23, 1.14)	.003
≥4	179	0.73 (0.09, 1.37)	.025	0.38 (−0.09, 0.84)	.11
(*p* value for linear trend)			(<.001)		(.007)
**Mothers’ Mm-MAST**	904				
0^a^	427	0		0	
1	201	−0.11 (−0.61, 0.40)	.68	0.08 (−0.28, 0.44)	.66
2	132	0.15 (−0.42, 0.72)	.60	−0.08 (−0.50, 0.33)	.70
3	69	0.79 (0.06, 1.51)	.033	0.39 (−0.22, 0.99)	.21
≥4	75	0.75 (−0.03, 1.54)	.061	0.24 (−0.32, 0.80)	.41
(*p* value for linear trend)			(.02)		(.33)
**Sons’ Mm-MAST at mid-twenties (per point increase)**				0.64 (0.59, 0.69)	<.001
**DAUGHTERS**					
**Fathers’ Mm-MAST**	1199				
0^a^	328	0		0	
1	245	0.14 (−0.30, 0.58)	.53	0.11 (−0.21, 0.44)	.48
2	228	0.15 (−0.30, 0.60)	.51	0.16 (−0.18, 0.50)	.36
3	161	0.82 (0.25, 1.40)	.005	0.49 (0.06, 0.93)	.026
≥4	237	0.98 (0.46, 1.49)	.000	0.73 (0.35, 1.12)	.000
(*p* value for linear trend)			(<.001)		(<.001)
**Mothers’ Mm-MAST**	1199				
0^a^	523	0		0	
1	277	−0.03 (−0.42, 0.37)	.90	−0.10 (−0.40, 0.20)	.51
2	182	0.12 (−0.39, 0.63)	.64	0.02 (−0.36, 0.40)	.91
3	110	0.17 (−0.39, 0.74)	.55	−0.17 (−0.61, 0.26)	.44
≥4	107	0.47 (−0.15, 1.09)	.14	0.25 (−0.20, 0.69)	.27
(*p* value for linear trend)			(.14)		(.62)
**Daughters’ Mm-MAST at mid-twenties (per point increase)**				0.64 (0.59, 0.69)	<.001

Note: Model 2 includes simultaneously fathers’ and mothers’ Mm-MAST + adjustments for fathers’ religiosity, mothers’ religiosity, fathers’ personality, mothers’ personality, fathers’ education, mothers’ education, area of residence, and family structure. Model 3 is as model 2 but additionally adjusted for offspring problem drinking (Mm-MAST-11) at mid-twenties. Lifetime abstainers excluded.

a Reference category

b *p* value for a difference with the reference category.

CI, confidence interval; Mm-MAST, Malmö-modified Michigan Alcoholism Screening Test (original 9-item version); Mm-MAST-11, Malmö-modified Michigan Alcoholism Screening Test (11-item version).

## References

[R1] Alati R, Baker P, Betts KS, Connor JP, Little K, Sanson A, & Olsson CA (2014). The role of parental alcohol use, parental discipline and antisocial behaviour on adolescent drinking trajectories. Drug and Alcohol Dependence, 134, 178–184. 10.1016/j.drugalcdep.2013.09.030 24479151

[R2] Anda RF, Whitfield CL, Felitti VJ, Chapman D, Edwards VJ, Dube SR, & Williamson DF (2002). Adverse childhood experiences, alcoholic parents, and later risk of alcoholism and depression. Psychiatric Services (Washington, D.C.), 53, 1001–1009. 10.1176/appi.ps.53.8.1001 12161676

[R3] Boden JM, Newton-Howes G, Foulds J, Spittlehouse J, & Cook S (2019). Trajectories of alcohol use problems based on early adolescent alcohol use: Findings from a 35 year population cohort. The International Journal on Drug Policy, 74, 18–25. 10.1016/j.drugpo.2019.06.011 31408802

[R4] Cameron AC, & Trivedi PK (2010). Microeconometrics using Stata (rev. ed.). Stata Press.

[R5] Connor JP, Haber PS, & Hall WD (2016). Alcohol use disorders. The Lancet, 387, 988–998. 10.1016/S0140-6736(15)00122-1 26343838

[R6] Cotton NS (1979). The familial incidence of alcoholism: A review. Journal of Studies on Alcohol, 40, 89–116. 10.15288/jsa.1979.40.89 376949

[R7] Dawson DA, Goldstein RB, Pickering RP, & Grant BF (2014). Nonresponse bias in survey estimates of alcohol consumption and its association with harm. Journal of Studies on Alcohol and Drugs, 75, 695–703. 24988268 10.15288/jsad.2014.75.695 PMC4108608

[R8] Delucchi KL, Matzger H, & Weisner C (2008). Alcohol in emerging adulthood: 7-year study of problem and dependent drinkers. Addictive Behaviors, 33, 134–142. 10.1016/j.addbeh.2007.04.027 17537582 PMC2100394

[R9] Edlund MJ, Harris KM, Koenig HG, Han X, Sullivan G, Mattox R, & Tang L (2010). Religiosity and decreased risk of substance use disorders: Is the effect mediated by social support or mental health status? Social Psychiatry and Psychiatric Epidemiology, 45, 827–836. 10.1007/s00127-009-0124-3 19714282 PMC2901801

[R10] Englund MM, Egeland B, Oliva EM, & Collins WA (2008). Childhood and adolescent predictors of heavy drinking and alcohol use disorders in early adulthood: A longitudinal developmental analysis. Addiction, 103, 23–35. 10.1111/j.1360-0443.2008.02174.x 18426538 PMC2822999

[R11] Gauffin K, Hemmingsson T, & Hjern A (2013). The effect of childhood socioeconomic position on alcohol-related disorders later in life: A Swedish national cohort study. Journal of Epidemiology and Community Health, 67, 932–938. 10.1136/jech-2013-202624 23814272

[R12] Gmel G, Kuntsche E, & Rehm J (2011). Risky single-occasion drinking: Bingeing is not bingeing. Addiction, 106, 1037–1045. 10.111/j.1360-0443.2010.03167.x 21564366

[R13] Grant BF, Goldstein RB, Saha TD, Chou SP, Jung J, Zhang H, Pickering RP, Ruan WJ, Smith SM, Huang B, & Hasin DS (2015). Epidemiology of DSM-5 Alcohol Use Disorder: Results From the National Epidemiologic Survey on Alcohol and Related Conditions III. JAMA Psychiatry, 72, 757–766. 10.1001/jamapsychiatry.2015.0584 26039070 PMC5240584

[R14] Harrell FE (2015). Regression modeling strategies: With applications to linear models, logistic regression, and survival analysis (2nd ed.). Springer.

[R15] Hemmingsson T, Danielsson A-K, & Falkstedt D (2017). Fathers’ alcohol consumption and risk of alcohol-related hospitalization in offspring before 60 years of age. Drugs: Education, Prevention and Policy, 24, 3–8. 10.1080/09687637.2016.1186154

[R16] Hopfer CJ, Crowley TJ, & Hewitt JK (2003). Review of twin and adoption studies of adolescent substance use. Journal of the American Academy of Child and Adolescent Psychiatry, 42, 710–719. 10.1097/01.CHI.0000046848.56865.54 12921479

[R17] Jacob T, Waterman B, Heath A, True W, Bucholz KK, Haber R, Scherrer J, & Fu Q (2003). Genetic and environmental effects on offspring alcoholism: New insights using an offspring-of-twins design. Archives of General Psychiatry, 60, 1265–1272. 10.1001/archpsyc.60.12.1265 14662559

[R18] Kaidesoja M, Aaltonen S, Bogl LH, Heikkilä K, Kaartinen S, Kujala UM, Kärkkäinen U, Masip G, Mustelin L, Palviainen T, Pietiläinen KH, Rottensteiner M, Sipilä PN, Rose RJ, Keski-Rahkonen A, & Kaprio J (2019). FinnTwin16: A longitudinal study from age 16 of a population-based Finnish twin cohort. Twin Research and Human Genetics, 22, 530–539. 10.1017/thg.2019.106 31796134

[R19] Kaprio J, Pulkkinen L, & Rose RJ (2002). Genetic and environmental factors in health-related behaviors: Studies on Finnish twins and twin families. Twin Research, 5, 366–371. 10.1375/twin.5.5.366 12537860

[R20] Kendler KS, Ji J, Edwards AC, Ohlsson H, Sundquist J, & Sundquist K (2015). An Extended Swedish National Adoption Study of Alcohol Use Disorder. JAMA Psychiatry, 72, 211. 10.1001/jamapsychiatry.2014.2138 25565339 PMC4351126

[R21] Kendler KS, Ohlsson H, Sundquist J, & Sundquist K (2015). Triparental families: A new genetic-epidemiological design applied to drug abuse, alcohol use disorders, and criminal behavior in a Swedish national sample. American Journal of Psychiatry, 172, 553–560. 10.1176/appi.ajp.2014.14091127 25698436 PMC4451407

[R22] Kendler KS, Prescott CA, Myers J, & Neale MC (2003). The structure of genetic and environmental risk factors for common psychiatric and substance use disorders in men and women. Archives of General Psychiatry, 60, 929–937. 10.1001/archpsyc.60.9.929 12963675

[R23] Kestilä L, Martelin T, Rahkonen O, Joutsenniemi K, Pirkola S, Poikolainen K, & Koskinen S (2008). Childhood and current determinants of heavy drinking in early adulthood. Alcohol and Alcoholism, 43, 460–469. 10.1093/alcalc/agn018 18364362

[R24] Knott CS, Bell S, & Britton A (2018). The stability of baseline-defined categories of alcohol consumption during the adult life-course: A 28-year prospective cohort study. Addiction, 113, 34–43. 10.1111/add.13949 28734088 PMC5725237

[R25] Kolevnikov S, & Angeles G (2004). The use of discrete data in PCA: theory, simulations, and applications to socioeconomic indices Carolina Population Center, University of North Carolina.

[R26] Kristenson H, & Trell E (1982). Indicators of alcohol consumption: Comparisons between a questionnaire (Mm-MAST), interviews and serum gamma-glutamyl transferase (GGT) in a health survey of middle-aged males. British Journal of Addiction, 77, 297–304. 6128016 10.1111/j.1360-0443.1982.tb02459.x

[R27] Landberg J, Danielsson A-K, Falkstedt D, & Hemmingsson T (2018). Fathers’ alcohol consumption and long-term risk for mortality in offspring. Alcohol and Alcoholism, 53, 753–759. 10.1093/alcalc/agy058 30137197 PMC6203123

[R28] Latendresse SJ, Rose RJ, Viken RJ, Pulkkinen L, Kaprio J, & Dick DM (2008). Parenting mechanisms in links between parents’ and adolescents’ alcohol use behaviors. Alcoholism: Clinical and Experimental Research, 32, 322–330. 10.1111/j.1530-0277.2007.00583.x 18162066 PMC2504716

[R29] Leonard KE, & Eiden RD (2007). Marital and family processes in the context of alcohol use and alcohol disorders. Annual Review of Clinical Psychology, 3, 285–310. 10.1146/annurev.clinpsy.3.022806.091424 PMC2667243 17716057

[R30] Leonard KE, & Rothbard JC (1999). Alcohol and the marriage effect. Journal of Studies on Alcohol. Supplement, 13, 139–146. 10225498 10.15288/jsas.1999.s13.139

[R31] Liu M, Jiang Y, Wedow R, Li Y, Brazel DM, Chen F, Datta G, Davila-Velderrain J, McGuire D, Tian C, Zhan X, 23andMe Research Team, HUNT All-In Psychiatry, Choquet H, Docherty AR, Faul JD, Foerster JR, Fritsche LG, Gabrielsen ME, … Vrieze S (2019). Association studies of up to 1.2 million individuals yield new insights into the genetic etiology of tobacco and alcohol use. Nature Genetics, 51, 237–244. 10.1038/s41588-018-0307-5 30643251 PMC6358542

[R32] Maes HH, Woodard CE, Murrelle L, Meyer JM, Silberg JL, Hewitt JK, Rutter M, Simonoff E, Pickles A, Carbonneau R, Neale MC, & Eaves LJ (1999). Tobacco, alcohol and drug use in eight- to sixteen-year-old twins: The Virginia Twin Study of Adolescent Behavioral Development. Journal of Studies on Alcohol, 60, 293–305. 10.15288/jsa.1999.60.293 10371255

[R33] Maggs JL, & Schulenberg JE (2004). Trajectories of alcohol use during the transition to adulthood. Alcohol Research & Health, 28, 195–201.

[R34] Maggs JL, & Staff JA (2018). Parents who allow early adolescents to drink. The Journal of Adolescent Health, 62, 245–247. 10.1016/j.jadohealth.2017.09.016 29254647

[R35] Mahedy L, MacArthur GJ, Hammerton G, Edwards AC, Kendler KS, Macleod J, Hickman M, Moore SC, & Heron J (2018). The effect of parental drinking on alcohol use in young adults: The mediating role of parental monitoring and peer deviance. Addiction, 113, 2041–2050. 10.1111/add.14280 29806869 PMC6176713

[R36] Mares SHW, van der Vorst H, Engels RCME, & Lichtwarck-Aschoff A (2011). Parental alcohol use, alcohol-related problems, and alcohol-specific attitudes, alcohol-specific communication, and adolescent excessive alcohol use and alcohol-related problems: An indirect path model. Addictive Behaviors, 36, 209–216. 10.1016/j.addbeh.2010.10.013 21084165

[R37] Mattick RP, Clare PJ, Aiken A, Wadolowski M, Hutchinson D, Najman J, Slade T, Bruno R, McBride N, Kypri K, Vogl L, & Degenhardt L (2018). Association of parental supply of alcohol with adolescent drinking, alcohol-related harms, and alcohol use disorder symptoms: A prospective cohort study. The Lancet Public Health, 3, e64–e71. 10.1016/S2468-2667(17)30240-2 29396259

[R38] McGovern R, Bogowicz P, Meader N, Kaner E, Alderson H, Craig D, Geijer-Simpson E, Jackson K, Muir C, Salonen D, Smart D, & Newham JJ (2023). The association between maternal and paternal substance use and child substance use, internalizing and externalizing problems: A systematic review and meta-analysis. Addiction. Advance online publication. 10.1111/add.16127 36607011

[R39] Merline A, Jager J, & Schulenberg JE (2008). Adolescent risk factors for adult alcohol use and abuse: Stability and change of predictive value across early and middle adulthood. Addiction, 103, 84–99. 10.1111/j.1360-0443.2008.02178.x 18426542 PMC2649657

[R40] Mitchell AJ, Meader N, Bird V, & Rizzo M (2012). Clinical recognition and recording of alcohol disorders by clinicians in primary and secondary care: Meta-analysis. The British Journal of Psychiatry, 201, 93–100. 10.1192/bjp.bp.110.091199 22859576

[R41] Mustanski BS, Viken RJ, Kaprio J, & Rose RJ (2003). Genetic influences on the association between personality risk factors and alcohol use and abuse. Journal of Abnormal Psychology, 112, 282–289. 12784838 10.1037/0021-843x.112.2.282

[R42] Northcote J, & Livingston M (2011). Accuracy of self-reported drinking: observational verification of ‘last occasion’ drink estimates of young adults. Alcohol and Alcoholism, 46, 709–713. 10.1093/alcalc/agr138 21949190

[R43] Nyström M, Peräsalo J, & Salaspuro M (1993). Screening for heavy drinking and alcohol-related problems in young university students: The CAGE, the Mm-MAST and the trauma score questionnaires. Journal of Studies on Alcohol, 54, 528–533. 10.15288/jsa.1993.54.528 8412142

[R44] Parra GR, Patwardhan I, Mason WA, Chmelka MB, Savolainen J, Miettunen J, & Järvelin M-R (2020). Parental alcohol use and the alcohol misuse of their offspring in a Finnish birth cohort: Investigation of developmental timing. Journal of Youth and Adolescence, 49, 1702–1715. 10.1007/s10964-020-01239-5 32378014

[R45] Pedersen W, & von Soest T (2013). Socialization to binge drinking: A population-based, longitudinal study with emphasis on parental influences. Drug and Alcohol Dependence, 133, 587–592. 10.1016/j.drugalcdep.2013.07.028 23993083

[R46] Pitkänen T, Kokko K, Lyyra A-L, & Pulkkinen L (2008). A developmental approach to alcohol drinking behaviour in adulthood: A follow-up study from age 8 to age 42. Addiction, 103, 48–68. 10.1111/j.1360-0443.2008.02176.x 18426540

[R47] Rehm J, Gmel GE, Gmel G, Hasan OSM, Imtiaz S, Popova S, Probst C, Roerecke M, Room R, Samokhvalov AV, Shield KD, & Shuper PA (2017). The relationship between different dimensions of alcohol use and the burden of disease-an update. Addiction, 112, 968–1001. 10.1111/add.13757 28220587 PMC5434904

[R48] Rose RJ, Dick DM, Viken RJ, & Kaprio J (2001). Gene-environment interaction in patterns of adolescent drinking: Regional residency moderates longitudinal influences on alcohol use. Alcoholism:Clinical and Experimental Research, 25, 637–643. 10.1111/j.1530-0277.2001.tb02261.x 11371711

[R49] Rose RJ, Kaprio J, Winter T, Koskenvuo M, & Viken RJ (1999). Familial and socioregional environmental effects on abstinence from alcohol at age sixteen. Journal of Studies on Alcohol. Supplement, 13, 63–74. 10225489 10.15288/jsas.1999.s13.63

[R50] Rossow I, Felix L, Keating P, & McCambridge J (2016). Parental drinking and adverse outcomes in children: A scoping review of cohort studies. Drug and Alcohol Review, 35, 397–405. 10.1111/dar.12319 26332090 PMC4950034

[R51] Rossow I, Keating P, Felix L, & McCambridge J (2016). Does parental drinking influence children’s drinking? A systematic review of prospective cohort studies. Addiction, 111, 204–217. 10.1111/add.13097 26283063 PMC4832292

[R52] Ruby JG, Wright KM, Rand KA, Kermany A, Noto K, Curtis D, Varner N, Garrigan D, Slinkov D, Dorfman I, Granka JM, Byrnes J, Myres N, & Ball C (2018). Estimates of the heritability of human longevity are substantially inflated due to assortative mating. Genetics, 210, 1109–1124. 10.1534/genetics.118.301613 30401766 PMC6218226

[R53] Schuckit MA, & Smith TL (1996). An 8-year follow-up of 450 sons of alcoholic and control subjects. Archives of General Psychiatry, 53, 202–210. 10.1001/archpsyc.1996.01830030020005 8611056

[R54] Seppä K, Koivula T, & Sillanaukee P (1992). Drinking habits and detection of heavy drinking among middle-aged women. Addiction, 87, 1703–1709. 10.1111/j.1360-0443.1992.tb02683.x 1490084

[R55] Seppä K, Sillanaukee P, & Koivula T (1990). The efficiency of a questionnaire in detecting heavy drinkers. Addiction, 85, 1639–1645. 10.1111/j.1360-0443.1990.tb01654.x 2289065

[R56] Sharmin S, Kypri K, Khanam M, Wadolowski M, Bruno R, Attia J, Holliday E, Palazzi K, & Mattick RP (2017). Effects of parental alcohol rules on risky drinking and related problems in adolescence: Systematic review and meta-analysis. Drug and Alcohol Dependence, 178, 243–256. 10.1016/j.drugalcdep.2017.05.011 28667942

[R57] Sher KJ, Walitzer KS, Wood PK, & Brent EE (1991). Characteristics of children of alcoholics: Putative risk factors, substance use and abuse, and psychopathology. Journal of Abnormal Psychology, 100, 427–448. 10.1037//0021-843x.100.4.427 1757657

[R58] Simpura J, & Lahti M-L (1988). Juomatapojen alueellinen vaihtelu Suomessa vuonna 1984 [The regional variation of drinking habits in Finland in 1984]. Alkoholipolitiikka, 53, 114–121.

[R59] Sipilä P, Harrasova G, Mustelin L, Rose RJ, Kaprio J, & Keski-Rahkonen A (2017). ‘Holy anorexia’-relevant or relic? Religiosity and anorexia nervosa among Finnish women. The International Journal of Eating Disorders, 50, 406–414. 10.1002/eat.22698 28346694

[R60] Sipilä P, Rose RJ, & Kaprio J (2016). Drinking and mortality: Long-term follow-up of drinking-discordant twin pairs. Addiction, 111, 245–254. 10.1111/add.13152 26359785 PMC4835818

[R61] Statistics Finland. (1998). Alueluokitukset = Regionala indelningar = Regional classifications handbook. Kunnat 1998 = Kommunerna 1998 = Municipalities 1998. Tilastokeskus [Statistics Finland].

[R62] Stone AL, Becker LG, Huber AM, & Catalano RF (2012). Review of risk and protective factors of substance use and problem use in emerging adulthood. Addictive Behaviors, 37, 747–775. 10.1016/j.addbeh.2012.02.014 22445418

[R63] Verhulst B, Neale MC, & Kendler KS (2015). The heritability of alcohol use disorders: A meta-analysis of twin and adoption studies. Psychological Medicine, 45, 1061–1072. 10.1017/S0033291714002165 25171596 PMC4345133

[R64] Viken RJ, Kaprio J, & Rose RJ (2007). Personality at ages 16 and 17 and drinking problems at ages 18 and 25: Genetic analyses of data from FinnTwin 16–25. Twin Research and Human Genetics, 10, 25–32. 10.1375/twin.10.1.25 17539362 PMC1993892

[R65] Walters RK, Polimanti R, Johnson EC, McClintick JN, Adams MJ, Adkins AE, Aliev F, Bacanu S-A, Batzler A, Bertelsen S, Biernacka JM, Bigdeli TB, Chen L-S, Clarke T-K, Chou Y-L, Degenhardt F, Docherty AR, Edwards AC, Fontanillas P, … 23andMe Research Team. (2018). Transancestral GWAS of alcohol dependence reveals common genetic underpinnings with psychiatric disorders. Nature Neuroscience, 21, 1656–1669. 10.1038/s41593-018-0275-1 30482948 PMC6430207

[R66] White HR, Johnson V, & Buyske S (2000). Parental modeling and parenting behavior effects on offspring alcohol and cigarette use. A growth curve analysis. Journal of Substance Abuse, 12, 287–310. 11367605 10.1016/s0899-3289(00)00056-0

[R67] Wiggins JS (1966). Substantive dimensions of self-report in the MMPI item pool. Psychological Monographs, 80, 1–42. 10.1037/h0093901 4381983

[R68] Williams RL (2000). A note on robust variance estimation for cluster-correlated data. Biometrics, 56, 645–646. 10877330 10.1111/j.0006-341x.2000.00645.x

[R69] Winter T, Karvonen S, & Rose RJ (2002). Does religiousness explain regional differences in alcohol use in Finland? Alcohol and Alcoholism, 37, 330–339. 12107034 10.1093/alcalc/37.4.330

[R70] Wooldridge JM (2010). Econometric analysis of cross section and panel data (2nd ed.). MIT Press.

[R71] Yap MBH, Cheong TWK, Zaravinos-Tsakos F, Lubman DI, & Jorm AF (2017). Modifiable parenting factors associated with adolescent alcohol misuse: A systematic review and meta-analysis of longitudinal studies. Addiction, 112, 1142–1162. 10.1111/add.13785 28178373

