# Supplementary material for: Paternal and Maternal Problem Drinking and Lifetime Problem Drinking of Their Adult Children
Source: Twin Res Hum Genet. Author manuscript; Available in PMC 2024 Apr 24. (PMC11000696; doi:10.1017/thg.2023.12)

Supplementary material

to Sipilä PN et al. Paternal and Maternal Problem Drinking
and Lifetime Problem Drinking of Their Adult Children

Table of contents

Supplementary Methods. 2
Supplementary Table 1 3
Supplementary Table 2 4
Supplementary Table 3 5
Supplementary Table 4 6
Supplementary Table 5 7
Supplementary Table 6 8
Supplementary Table 7 9
Supplementary Table 8 10
Supplementary Figure 1 11
Supplementary Figure 2 12
Supplementary Figure 3 13
Supplementary Figure 4 14
Supplementary Figure 5 15

Supplementary Methods. Supplementary information on measures of problem drinking, heavy drinking occasions and covariates.

Problem drinking (Mm-MAST and Mm-MAST-11)

For both measures of problem drinking, Mm-MAST and Mm-MAST-11, we included all responses with no more than two missing items, and substituted for the missing items the mean score of the available items of each included respondent. Substitutions were done for 6% of fathers and mothers and 1% of their adult children. Further, in wave 1, respondent parents were instructed to skip the entire scale if they did not drink at all. Therefore, fathers and mothers received an Mm-MAST score of zero if all items were missing and the respondent did not drink alcohol during the past year, but did not self-attribute lifetime abstinence. Fathers and mothers, as well as their sons and daughters, were excluded from the analyses if they were lifetime abstainers.

Among all who returned questionnaires, 79% of fathers had answered all 9 items, 85% had at most two missing items, 5% did not drink during the past year and 7% were lifetime abstainers; similarly, 73% of mothers had answered all 9 items, 79% had at most two missing items, 7% did not drink during the past year and 13% were lifetime abstainers. At mid-twenties and mid-thirties, 98% and 94% of sons had answered all 11 items, 99% and 95% had at most two missing items and 4% and 2% were lifetime abstainers, respectively. For daughters at mid-twenties and mid-thirties, corresponding percentages were 98% and 95%, 99% and 95%, and 4% and 3%, respectively.

Fathers' and mothers' religiosity

Among those who returned the questionnaire, all 12 items of the Religious fundamentalism content scale of the Minnesota Multiphasic Personality Inventory (MMPI) were answered by 74% of fathers and 73% of mothers, and 91% of fathers and 93% of mothers had answered at least 9 items. We included those answering at least 9 items, and mean score of the available items of each particular individual was substituted for missing items.

Fathers' and mothers' personality

Among those who returned the questionnaire, all 50 items of the social deviance scale (Pd or "Psychopathic deviate" scale of the MMPI) were answered by 74% of fathers and 73% of mothers, and 96% of fathers and 97% of mothers had answered at least 40 items. Therefore, we included those answering at least 40 items, and mean score of the available items of each particular individual was substituted for missing items.

Supplementary Table 1. Background characteristics of the participants by inclusion to versus exclusion from the mid-twenties main analysis

	Men		Women	
	Included (n=1,235)	Excluded (n=1,987)	Included (n=1,461)	Excluded (n=1,821)
Father's religiosity	3.4 (2.8)	4.5 (3.5)	3.3 (2.7)	4.8 (3.5)
Mother's religiosity	4.6 (2.7)	5.5 (3.0)	4.6 (2.7)	5.8 (3.1)
Father's personality (Pd scale)	15.8 (4.9)	15.7 (5.2)	16.1 (5.1)	15.9 (5.5)
Mother's personality (Pd scale)	15.8 (4.9)	15.7 (5.2)	16.1 (5.1)	15.9 (5.5)
Father's problem drinking				
0	322 (26.1%)	422 (21.2%)	391 (26.8%)	390 (21.4%)
1	243 (19.7%)	170 (8.6%)	294 (20.1%)	154 (8.5%)
2	218 (17.7%)	134 (6.7%)	276 (18.9%)	137 (7.5%)
3	195 (15.8%)	90 (4.5%)	194 (13.3%)	97 (5.3%)
≥4	257 (20.8%)	194 (9.8%)	306 (20.9%)	131 (7.2%)
(missing)	0 (0.0%)	977 (49.2%)	0 (0.0%)	912 (50.1%)
Mother's problem drinking				
0	572 (46.3%)	735 (37.0%)	649 (44.4%)	734 (40.3%)
1	285 (23.1%)	217 (10.9%)	327 (22.4%)	190 (10.4%)
2	180 (14.6%)	120 (6.0%)	222 (15.2%)	90 (4.9%)
3	92 (7.4%)	79 (4.0%)	134 (9.2%)	67 (3.7%)
≥4	106 (8.6%)	92 (4.6%)	129 (8.8%)	78 (4.3%)
(missing)	0 (0.0%)	744 (37.4%)	0 (0.0%)	662 (36.4%)
Father's heavy drinking occasions				
never	185 (15.0%)	344 (17.3%)	245 (16.8%)	360 (19.8%)
once a year or less often	173 (14.0%)	104 (5.2%)	170 (11.6%)	83 (4.6%)
a few times a year	376 (30.4%)	261 (13.1%)	453 (31.0%)	215 (11.8%)
about once a month	237 (19.2%)	157 (7.9%)	267 (18.3%)	141 (7.7%)
about once a week or more often	259 (21.0%)	193 (9.7%)	317 (21.7%)	159 (8.7%)
(missing)	5 (0.4%)	928 (46.7%)	9 (0.6%)	863 (47.4%)
Mother's heavy drinking occasions				
never	604 (48.9%)	793 (39.9%)	700 (47.9%)	779 (42.8%)
once a year or less often	207 (16.8%)	168 (8.5%)	247 (16.9%)	143 (7.9%)
a few times a year	249 (20.2%)	204 (10.3%)	301 (20.6%)	165 (9.1%)
about once a month	110 (8.9%)	81 (4.1%)	124 (8.5%)	81 (4.4%)
about once a week or more often	61 (4.9%)	63 (3.2%)	80 (5.5%)	45 (2.5%)
(missing)	4 (0.3%)	678 (34.1%)	9 (0.6%)	608 (33.4%)
Father's education				
academic	287 (23.2%)	156 (7.9%)	325 (22.2%)	143 (7.9%)
non-academic	948 (76.8%)	912 (45.9%)	1136 (77.8%)	830 (45.6%)
(missing)	0 (0.0%)	919 (46.3%)	0 (0.0%)	848 (46.6%)
Mother's education				
academic	357 (28.9%)	238 (12.0%)	428 (29.3%)	243 (13.3%)
non-academic	878 (71.1%)	1074 (54.1%)	1033 (70.7%)	982 (53.9%)
(missing)	0 (0.0%)	675 (34.0%)	0 (0.0%)	596 (32.7%)
Area of residence in adolescence				
capital area	312 (25.3%)	346 (17.4%)	356 (24.4%)	278 (15.3%)
Mid-Finland or West coast	175 (14.2%)	261 (13.1%)	206 (14.1%)	273 (15.0%)
rest of Finland	748 (60.6%)	989 (49.8%)	899 (61.5%)	1015 (55.7%)
(missing)	0 (0.0%)	391 (19.7%)	0 (0.0%)	255 (14.0%)
Living with both parents in adolescence				
Yes	1116 (90.4%)	1137 (57.2%)	183 (12.5%)	403 (22.1%)
No	119 (9.6%)	394 (19.8%)	1278 (87.5%)	1119 (61.4%)
(missing)	0 (0.0%)	456 (22.9%)	0 (0.0%)	299 (16.4%)

Data are mean (standard deviation) or N (%).

Supplementary Table 2. Background characteristics of the participants by inclusion to versus exclusion from the mid-thirties main analysis

	Men		Women	
	Included (n=991)	Excluded (n=2,231)	Included (n=1,278)	Excluded (n=2,004)
Father's religiosity	3.3 (2.7)	4.3 (3.4)	3.3 (2.7)	4.5 (3.4)
Mother's religiosity	4.5 (2.6)	5.4 (3.0)	4.6 (2.7)	5.7 (3.0)
Father's personality (Pd scale)	15.7 (4.9)	15.9 (5.1)	16.1 (5.1)	16.0 (5.4)
Mother's personality (Pd scale)	15.7 (4.9)	15.9 (5.1)	16.1 (5.1)	16.0 (5.4)
Father's problem drinking				
0	264 (26.6%)	480 (21.5%)	344 (26.9%)	437 (21.8%)
1	191 (19.3%)	222 (10.0%)	258 (20.2%)	190 (9.5%)
2	187 (18.9%)	165 (7.4%)	244 (19.1%)	169 (8.4%)
3	152 (15.3%)	133 (6.0%)	178 (13.9%)	113 (5.6%)
≥4	197 (19.9%)	254 (11.4%)	254 (19.9%)	183 (9.1%)
(missing)	0 (0.0%)	977 (43.8%)	0 (0.0%)	912 (45.5%)
Mother's problem drinking				
0	459 (46.3%)	848 (38.0%)	550 (43.0%)	833 (41.6%)
1	222 (22.4%)	280 (12.6%)	300 (23.5%)	217 (10.8%)
2	148 (14.9%)	152 (6.8%)	192 (15.0%)	120 (6.0%)
3	75 (7.6%)	96 (4.3%)	125 (9.8%)	76 (3.8%)
≥4	87 (8.8%)	111 (5.0%)	111 (8.7%)	96 (4.8%)
(missing)	0 (0.0%)	744 (33.3%)	0 (0.0%)	662 (33.0%)
Father's heavy drinking occasions				
never	145 (14.6%)	384 (17.2%)	226 (17.7%)	379 (18.9%)
once a year or less often	137 (13.8%)	140 (6.3%)	143 (11.2%)	110 (5.5%)
a few times a year	314 (31.7%)	323 (14.5%)	394 (30.8%)	274 (13.7%)
about once a month	176 (17.8%)	218 (9.8%)	229 (17.9%)	179 (8.9%)
about once a week or more often	215 (21.7%)	237 (10.6%)	278 (21.8%)	198 (9.9%)
(missing)	4 (0.4%)	929 (41.6%)	8 (0.6%)	864 (43.1%)
Mother's heavy drinking occasions				
never	489 (49.3%)	908 (40.7%)	612 (47.9%)	867 (43.3%)
once a year or less often	161 (16.2%)	214 (9.6%)	215 (16.8%)	175 (8.7%)
a few times a year	209 (21.1%)	244 (10.9%)	267 (20.9%)	199 (9.9%)
about once a month	79 (8.0%)	112 (5.0%)	106 (8.3%)	99 (4.9%)
about once a week or more often	49 (4.9%)	75 (3.4%)	73 (5.7%)	52 (2.6%)
(missing)	4 (0.4%)	678 (30.4%)	5 (0.4%)	612 (30.5%)
Father's education				
academic	246 (24.8%)	197 (8.8%)	301 (23.6%)	167 (8.3%)
non-academic	745 (75.2%)	1115 (50.0%)	977 (76.4%)	989 (49.4%)
(missing)	0 (0.0%)	919 (41.2%)	0 (0.0%)	848 (42.3%)
Mother's education				
academic	297 (30.0%)	298 (13.4%)	382 (29.9%)	289 (14.4%)
non-academic	694 (70.0%)	1258 (56.4%)	896 (70.1%)	1119 (55.8%)
(missing)	0 (0.0%)	675 (30.3%)	0 (0.0%)	596 (29.7%)
Area of residence in adolescence				
capital area	255 (25.7%)	403 (18.1%)	315 (24.6%)	319 (15.9%)
Mid-Finland or West coast	140 (14.1%)	296 (13.3%)	188 (14.7%)	291 (14.5%)
rest of Finland	596 (60.1%)	1141 (51.1%)	775 (60.6%)	1139 (56.8%)
(missing)	0 (0.0%)	391 (17.5%)	0 (0.0%)	255 (12.7%)
Living with both parents in adolescence				
Yes	890 (89.8%)	1363 (61.1%)	1111 (86.9%)	1286 (64.2%)
No	101 (10.2%)	412 (18.5%)	167 (13.1%)	419 (20.9%)
(missing)	0 (0.0%)	456 (20.4%)	0 (0.0%)	299 (14.9%)

Data are mean (standard deviation) or N (%).

Supplementary Table 3. Items of the Malmö-modified Michigan Alcoholism Screening Test in the study

Mm-MAST items for fathers and mothers

1. Do you take a drink before going to a party?
2. Do you usually drink a bottle of wine or corresponding amount of beer or other alcoholic beverages over the weekend?
3. Do you drink a couple of drinks (or beers) a day to relax?
4. Do you tolerate more alcohol now than you did ten years ago?
5. Have you difficulties not drinking more than your friends?
6. Do you fall asleep after moderate drinking without knowing how you got to bed?
7. Do you have a bad conscience after drinking?
8. Do you take a drink (the day after a party) for your hang-over?
9. Do you try to avoid alcoholic beverages for a determined period of time – e.g., a week?

Mm-MAST-11 items for sons and daughters at mid-twenties and mid-thirties

1. Do/did you take a drink before going to a party?
2. Do/did you usually drink a bottle of wine or corresponding amount of beer or other alcoholic beverages over the weekend?
3. Do/did you drink a couple of drinks (or beers) a day to relax?
4. Do/did you tolerate more alcohol now than before?
5. Have/had you difficulties not drinking more than your friends?
6. Do/did you fall asleep after moderate drinking without knowing how you got to bed?
7. Do/did you have a bad conscience after drinking?
8. Do/did you take a drink (the day after a party) for your hang-over?
9. Do/did you try to avoid alcoholic beverages for a determined period of time – e.g., a week?
10. *After you have/had taken a drink, do you find it hard to stop?
11. *Have/had you ever felt that anyone close to you thinks that you should drink less?

*Items not included in the original 9-item Mm-MAST. Abbreviations: Mm-MAST, Malmö-modified Michigan Alcoholism Screening Test (original 9-item version); Mm-MAST-11, Malmö-modified Michigan Alcoholism Screening Test (extended 11-item version)

Supplementary Table 4. Correlations between measures of problem drinking and covariates.

	1	2	3	4	5	6	7	8	9	10	11	12	13	14	15	16	17	18	19
1 Fathers’ Mm-Mast	1	0.40	0.18	0.12	0.16	0.18	0.71	0.32	0.16	0.15	0.07	0.15	-0.09	-0.08	0.27	0.15	-0.01	-0.02	-0.15
2 Mothers’ Mm-Mast		1	0.12	0.09	0.14	0.11	0.36	0.67	0.14	0.14	0.06	0.11	-0.08	-0.10	0.06	0.24	0.07	-0.01	-0.16
3 Sons’ lifetime Mm-MAST-11 at mid-twenties			1	0.13	0.65	0.15	0.16	0.17	0.57	0.08	0.41	0.23	-0.01	-0.05	0.10	0.04	0.08	0.06	-0.05
4 Daughters’ lifetime Mm-MAST-11 at mid-twenties				1	0.17	0.63	0.08	0.14	0.09	0.59	0.18	0.36	-0.02	0.00	0.07	0.05	0.06	0.04	-0.04
5 Sons’ lifetime Mm-MAST-11 at mid-thirties					1	0.22	0.11	0.18	0.51	0.11	0.50	0.24	-0.01	-0.01	0.05	0.05	0.13	0.07	-0.01
6 Daughters’ lifetime Mm-MAST-11 at mid-thirties						1	0.16	0.13	0.12	0.52	0.12	0.49	0.00	-0.01	0.09	0.07	0.00	0.02	-0.08
7 Fathers’ heavy drinking occasions							1	0.46	0.20	0.11	0.12	0.13	-0.17	-0.13	0.18	0.13	-0.02	-0.06	-0.12
8 Mothers’ heavy drinking occasions								1	0.15	0.17	0.15	0.12	-0.14	-0.23	0.09	0.25	-0.03	-0.11	-0.20
9 Sons’ heavy drinking occasions at mid-twenties									1	0.17	0.57	0.18	-0.03	-0.06	0.02	-0.02	0.06	0.05	-0.02
10 Daughters’ heavy drinking occasions at mid-twenties										1	0.20	0.53	-0.05	-0.06	0.04	0.05	0.08	0.04	-0.04
11 Sons’ heavy drinking occasions at mid-thirties											1	0.27	-0.01	-0.05	-0.04	-0.04	0.05	-0.01	0.02
12 Daughters’ heavy drinking occasions at mid-thirties												1	-0.04	-0.03	0.04	0.12	-0.03	-0.05	-0.02
13 Fathers' religiosity													1	0.43	0.04	0.04	-0.04	-0.01	0.05
14 Mothers' religiosity														1	0.02	0.02	-0.05	-0.06	0.11
15 Fathers' personality (Pd scale)															1	0.25	-0.13	-0.08	-0.25
16 Mothers' personality (Pd scale)																1	-0.10	-0.12	-0.22
17 Fathers' education																	1	0.72	0.07
18 Mothers' education																		1	0.02
19 Living with both parents in adolescence																			1

The reported correlations are tetrachoric correlations between binary variables, polychoric correlations between ordinal variables,
biserial correlations between a binary variable and a continuous variable, polyserial correlations between an ordinal variable and a continuous variable,
and Pearson correlation coefficients between continuous variables. Area of residence was not included in the correlation analysis, because it is a nominal variable.
Abbreviations: Mm-MAST, Malmö-modified Michigan Alcoholism Screening Test (original 9-item version);
Mm-MAST-11, Malmö-modified Michigan Alcoholism Screening Test (extended 11-item version).

Supplementary Table 5. Correlations between measures of problem drinking and heavy drinking occasions when the analysis was restricted to those who were living with both biological parents at age 16.

**POLYSERIAL CORRELATIONS**	Sons' lifetime Mm-MAST-11 at mid-twenties (n=1,116)	Daughters' lifetime Mm-MAST-11 at mid-twenties (n=1,278)	Sons' lifetime Mm-MAST-11 at mid-thirties (n=890)	Daughters' lifetime Mm-MAST-11 at mid-thirties (n=1,111)
Fathers' Mm-Mast	0.17**	0.13**	0.15**	0.19**
Mothers' Mm-Mast	0.11*	0.11*	0.12*	0.12**

**POLYCHORIC CORRELATIONS**	Sons' heavy drinking occasions at mid-twenties (n=1,155)	Daughters' heavy drinking occasions at mid-twenties (n=1,310)	Sons' heavy drinking occasions at mid-thirties (n=927)	Daughters' heavy drinking occasions at mid-thirties (n=1,149)
Fathers' heavy drinking occasions	0.21**[a] (rho = 0.19**)	0.14**[a] (rho = 0.12**)	0.13**	0.15**
Mothers' heavy drinking occasions	0.17**[a] (rho = 0.14**)	0.18**	0.18**[a] (rho = 0.15**)	0.14**

	Fathers' Mm-Mast (n=1,336)	Mothers' Mm-Mast (n=1,336)	Fathers' heavy drinking occasions (n=1,336)	Mothers' heavy drinking occasions (n=1,336)
Fathers' Mm-Mast	1	0.42**[a] (rho = 0.36**)	0.70**	0.35**
Mothers' Mm-Mast		1	0.39**[a] (rho = 0.34**)	0.67**
Fathers' heavy drinking occasions			1	0.52**[a] (rho = 0.43**)
Mothers' heavy drinking occasions				1

*p<0.01, **p<0.001. [a]Pearson's chi-squared test indicates violation of the underlying bivariate normality assumption. Spearman's rank order correlation coefficients (rho) given for comparison in parentheses for those polychoric correlations for which there was evidence for violation of the underlying bivariate normality assumption. Lifetime abstainers were excluded from the analysis. Abbreviations: Mm-MAST, Malmö-modified Michigan Alcoholism Screening Test (original 9-item version); Mm-MAST-11, Malmö-modified Michigan Alcoholism Screening Test (extended 11-item version).

Supplementary Table 6. Polyserial and polychoric correlations between measures of problem drinking and heavy drinking occasions with lifetime abstainers included.

**POLYSERIAL CORRELATIONS**	Sons' lifetime Mm-MAST-11 at mid-twenties (n=1,464)	Daughters' lifetime Mm-MAST-11 at mid-twenties (n=1,717)	Sons' lifetime Mm-MAST-11 at mid-thirties (n=1,130)	Daughters' lifetime Mm-MAST-11 at mid-thirties (n=1,453)
Fathers' Mm-Mast	0.24**	0.17**	0.19**	0.20**
Mothers' Mm-Mast	0.18**	0.15**	0.15**	0.13**

**POLYCHORIC CORRELATIONS**	Sons' heavy drinking occasions at mid-twenties (n=1,521)	Daughters' heavy drinking occasions at mid-twenties (n=1,774)	Sons' heavy drinking occasions at mid-thirties (n=1,203)	Daughters' heavy drinking occasions at mid-thirties (n=1,550)
Fathers' heavy drinking occasions	0.29**[a] (rho = 0.26**)	0.22**[a] (rho = 0.19**)	0.21**[a] (rho = 0.18**)	0.21**
Mothers' heavy drinking occasions	0.24**[a] (rho = 0.20**)	0.24**	0.20**[a] (rho = 0.17**)	0.18**[a] (rho = 0.16**)
	Fathers' Mm-Mast (n=1,771)	Mothers' Mm-Mast (n=1,771)	Fathers' heavy drinking occasions (n=1,771)	Mothers' heavy drinking occasions (n=1,771)
Fathers' Mm-Mast	1	0.45**[a] (rho = 0.38**)	0.75**[a] (rho = 0.69**)	0.38**
Mothers' Mm-Mast		1	0.43**[a] (rho = 0.36**)	0.72**
Fathers' heavy drinking occasions			1	0.52**[a] (rho = 0.43**)
Mothers' heavy drinking occasions				1

*p<0.01, **p<0.001. [a]Pearson's chi-squared test indicates violation of the underlying bivariate normality assumption. Spearman's rank order correlation coefficients (rho) given for comparison in parentheses for those polychoric correlations for which there was evidence for violation of the underlying bivariate normality assumption. Abbreviations: Mm-MAST, Malmö-modified Michigan Alcoholism Screening Test (original 9-item version); Mm-MAST-11, Malmö-modified Michigan Alcoholism Screening Test (extended 11-item version).

Supplementary Table 7. Association of fathers' and mothers' problem drinking with lifetime problem drinking of offspring at mid-twenties with lifetime abstainers included.

		MODEL 1 *(basic model)*		MODEL 2 *(multiply-adjusted model)*	
	N	β (95% CI)	*p*-value[b]	β (95% CI)	*p*-value[b]
**SONS**					
**Fathers' problem drinking (Mm-MAST)**	1,464				
0[a]	478	0		0	
1	262	0.76 (0.29, 1.23)	0.002	0.58 (0.10, 1.06)	0.017
2	238	1.18 (0.67, 1.68)	<0.001	0.92 (0.41, 1.44)	<0.001
3	202	1.33 (0.80, 1.86)	<0.001	1.11 (0.57, 1.65)	<0.001
≥4	284	1.39 (0.90, 1.88)	<0.001	1.13 (0.61, 1.64)	<0.001
(*p*-value for linear trend)			(<0.001)		(<0.001)
**Mothers' problem drinking (Mm-MAST)**	1,464				
0[a]	773	0		0	
1	303	-0.12 (-0.54, 0.30)	0.57	-0.21 (-0.63, 0.22)	0.34
2	189	0.42 (-0.08, 0.91)	0.10	0.34 (-0.16, 0.84)	0.18
3	92	0.81 (0.14, 1.49)	0.019	0.66 (-0.02, 1.33)	0.056
≥4	107	0.85 (0.15, 1.54)	0.017	0.66 (-0.06, 1.39)	0.073
(*p*-value for linear trend)			(0.002)		(0.016)
**DAUGHTERS**					
**Fathers' problem drinking (Mm-MAST)**	1,717				
0[a]	557	0		0	
1	324	0.53 (0.13, 0.94)	0.01	0.44 (0.03, 0.84)	0.036
2	300	0.35 (-0.03, 0.73)	0.073	0.24 (-0.15, 0.62)	0.23
3	209	0.79 (0.35, 1.23)	<0.001	0.66 (0.22, 1.10)	0.004
≥4	327	0.87 (0.48, 1.25)	<0.001	0.70 (0.30, 1.10)	0.001
(*p*-value for linear trend)			(<0.001)		(0.001)
**Mothers' problem drinking (Mm-MAST)**	1,717				
0[a]	889	0	0	0	
1	333	0.31 (-0.03, 0.65)	0.075	0.24 (-0.10, 0.59)	0.16
2	226	0.25 (-0.17, 0.67)	0.24	0.20 (-0.23, 0.62)	0.37
3	139	0.50 (0.03, 0.97)	0.038	0.43 (-0.04, 0.91)	0.073
≥4	130	0.65 (0.13, 1.16)	0.014	0.53 (0.00, 1.07)	0.052
(*p*-value for linear trend)			(0.004)		(0.021)

[a]Reference category
[b]*p*-value for a difference with the reference category
Model 1 includes simultaneously fathers' and mothers' Mm-MAST. Model 2 includes simultaneously fathers' and mothers' Mm-MAST + adjustments for fathers' religiosity, mothers' religiosity, fathers' personality, mothers' personality, fathers' education, mothers' education, area of residence, and family structure. Lifetime abstainers included. Offspring problem drinking measured using Mm-MAST-11. Abbreviations: CI, confidence interval; Mm-MAST, Malmö-modified Michigan Alcoholism Screening Test (original 9-item version); Mm-MAST-11, Malmö-modified Michigan Alcoholism Screening Test (extended 11-item version).

Supplementary Table 8. Association of fathers' and mothers' problem drinking with lifetime problem drinking of offspring at mid-thirties with lifetime abstainers included.

		MODEL 1 *(basic model)*		MODEL 2 *(multiply-adjusted model)*	
	N	β (95% CI)	*p*-value[b]	β (95% CI)	*p*-value[b]
**SONS**					
**Fathers' problem drinking (Mm-MAST)**	1,130				
0[a]	358	0		0	
1	206	0.11 (-0.41, 0.63)	0.67	0.01 (-0.50, 0.53)	0.97
2	198	0.81 (0.28, 1.33)	0.003	0.74 (0.22, 1.27)	0.006
3	156	1.21 (0.63, 1.80)	<0.001	1.20 (0.60, 1.80)	<0.001
≥4	212	0.85 (0.32, 1.38)	0.002	0.82 (0.26, 1.38)	0.004
(*p*-value for linear trend)			(<0.001)		(<0.001)
**Mothers' problem drinking (Mm-MAST)**	1,130				
0[a]	585	0		0	
1	230	0.04 (-0.43, 0.52)	0.86	0.04 (-0.43, 0.51)	0.87
2	153	0.07 (-0.44, 0.58)	0.79	0.06 (-0.45, 0.57)	0.81
3	75	0.93 (0.21, 1.64)	0.011	0.82 (0.10, 1.55)	0.027
≥4	87	0.85 (0.18, 1.53)	0.014	0.69 (-0.03, 1.41)	0.061
(*p*-value for linear trend)			(0.005)		(0.023)
**DAUGHTERS**					
**Fathers' problem drinking (Mm-MAST)**	1,453				
0[a]	458	0		0	
1	274	0.18 (-0.22, 0.58)	0.38	0.13 (-0.29, 0.54)	0.54
2	262	0.22 (-0.20, 0.63)	0.31	0.12 (-0.30, 0.54)	0.59
3	190	0.90 (0.38, 1.42)	0.001	0.80 (0.26, 1.33)	0.004
≥4	269	1.13 (0.67, 1.59)	<0.001	1.00 (0.51, 1.49)	<0.001
(*p*-value for linear trend)			(<0.001)		(<0.001)
**Mothers' problem drinking (Mm-MAST)**	1,453				
0[a]	720	0		0	
1	304	0.03 (-0.34, 0.41)	0.87	0.05 (-0.33, 0.42)	0.81
2	193	0.16 (-0.31, 0.64)	0.50	0.13 (-0.35, 0.61)	0.60
3	125	0.30 (-0.25, 0.85)	0.29	0.28 (-0.27, 0.83)	0.31
≥4	111	0.64 (0.04, 1.23)	0.036	0.55 (-0.05, 1.15)	0.073
(*p*-value for linear trend)			(0.034)		(0.067)

[a]Reference category
[b]*p*-value for a difference with the reference category
Model 1 includes simultaneously fathers' and mothers' Mm-MAST. Model 2 includes simultaneously fathers' and mothers' Mm-MAST + adjustments for fathers' religiosity, mothers' religiosity, fathers' personality, mothers' personality, fathers' education, mothers' education, area of residence, and family structure. Lifetime abstainers included. Abbreviations: CI, confidence interval; Mm-MAST, Malmö-modified Michigan Alcoholism Screening Test (original 9-item version); Mm-MAST-11, Malmö-modified Michigan Alcoholism Screening Test (extended 11-item version).

Supplementary Figure 1. Flowchart of the study cohort.

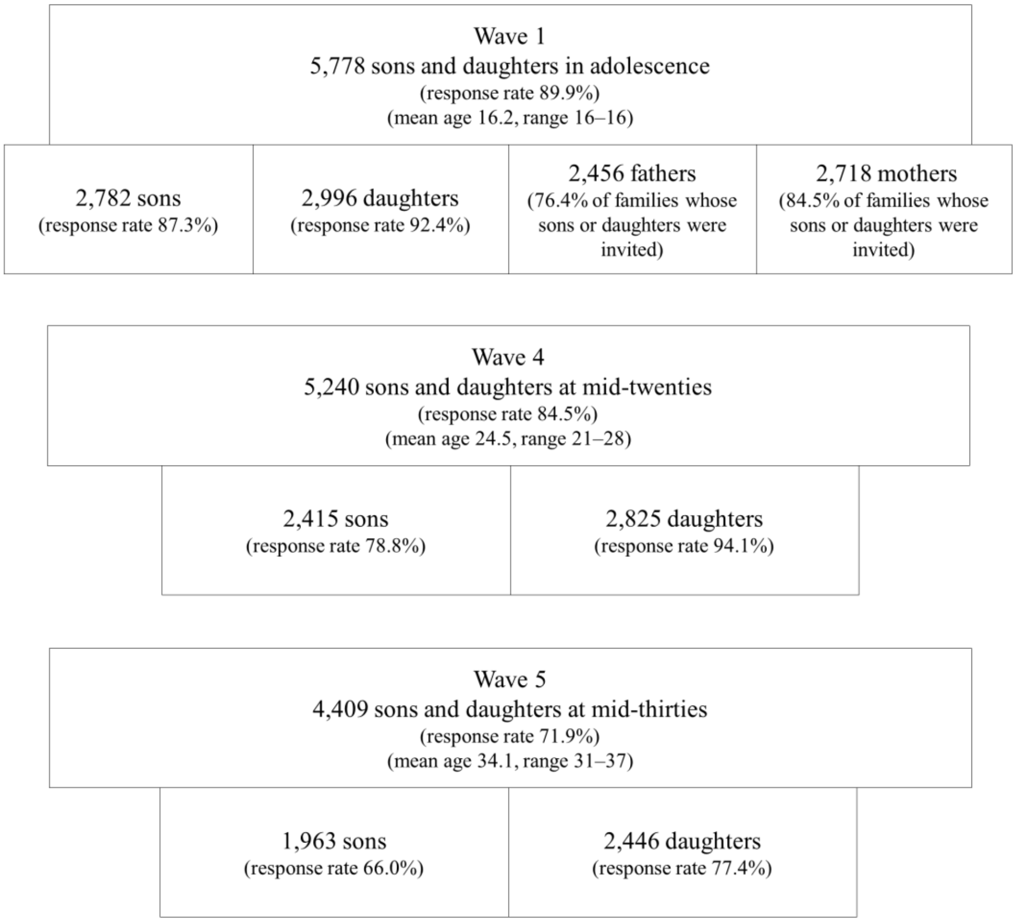

Supplementary Figure 2. Sons’ heavy drinking occasions by fathers’ heavy drinking occasions. Sons’ heavy drinking occasions as adjusted mean heavy drinking occasions per year (with 95% confidence intervals) at mid-twenties (21–28 years) and at mid-thirties (31–37 years). * = P < 0.05 for difference with baseline category where the father never has heavy drinking occasions. Fathers’ heavy drinking occasions measured when the sons were 16 years old. Estimates adjusted for area of residence, family structure, fathers’ and mothers’ education, religiosity and personality and mothers’ heavy drinking occasions. Lifetime abstainers excluded. The results are available in tabular form from the corresponding author upon request.

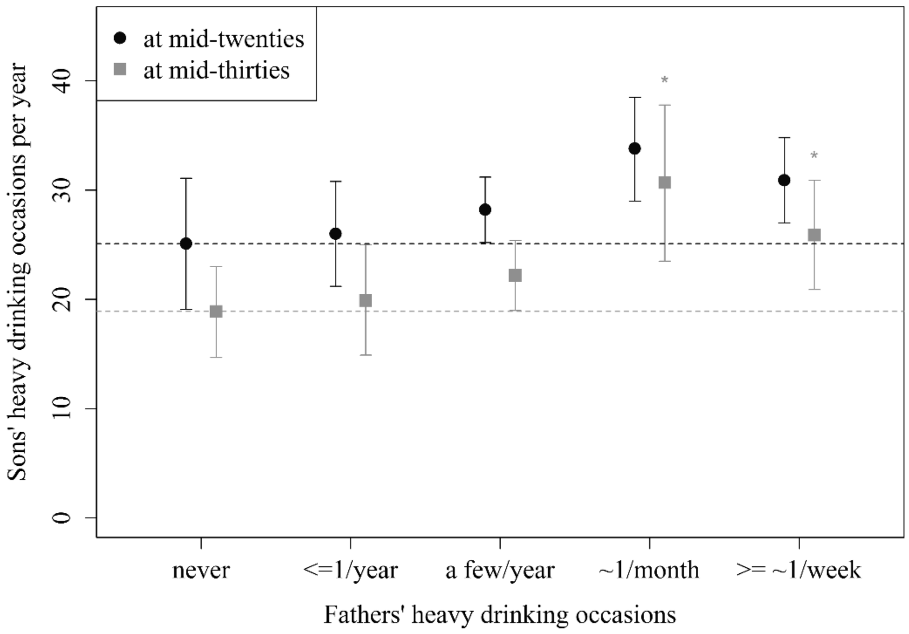

Supplementary Figure 3. Daughters’ heavy drinking occasions by fathers’ heavy drinking occasions. Daughters’ heavy drinking occasions as adjusted mean heavy drinking occasions per year (with 95% confidence intervals) at mid-twenties (21–28 years) and at mid-thirties (31–37 years). * = P < 0.05 for difference with baseline category where the father never has heavy drinking occasions. Fathers’ heavy drinking occasions measured when the daughters were 16 years old. Estimates adjusted for area of residence, family structure, fathers’ and mothers’ education, religiosity and personality and mothers’ heavy drinking occasions. Lifetime abstainers excluded. The results are available in tabular form from the corresponding author upon request.

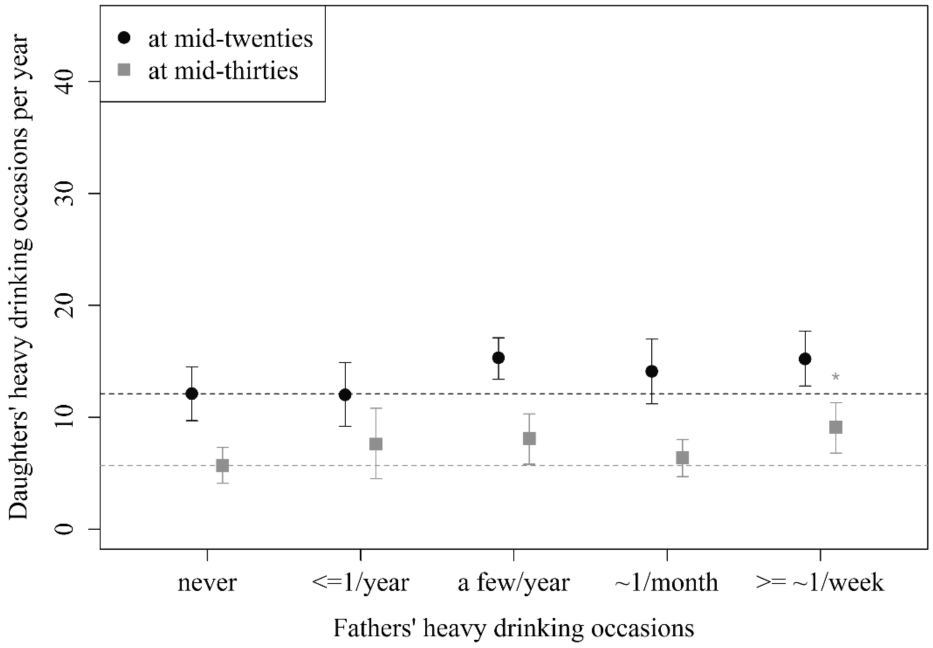

Supplementary Figure 4. Sons' heavy drinking occasions by mothers' heavy drinking occasions. Sons' heavy drinking occasions as adjusted mean heavy drinking occasions per year (with 95% confidence intervals) at mid-twenties (21–28 years) and at mid-thirties (31–37 years). * = P < 0.05 for difference with baseline category where the mother never has heavy drinking occasions. Mothers' heavy drinking occasions measured when the daughters were 16 years old. Estimates adjusted for area of residence, family structure, fathers' and mothers' education, religiosity and personality and fathers' heavy drinking occasions. Lifetime abstainers excluded. The results are available in tabular form from the corresponding author upon request.

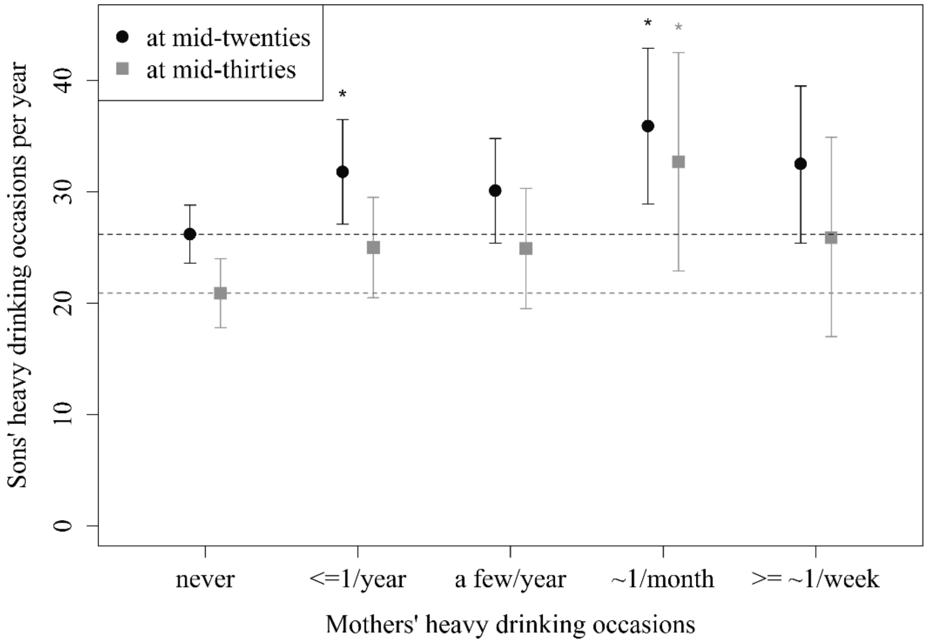

Supplementary Figure 5. Daughters' heavy drinking occasions by mothers' heavy drinking occasions. Daughters' heavy drinking occasions as adjusted mean heavy drinking occasions per year (with 95% confidence intervals) at mid-twenties (21–28 years) and at mid-thirties (31–37 years). * = P < 0.05 for difference with baseline category where the mother never has heavy drinking occasions. Mothers' heavy drinking occasions measured when the daughters were 16 years old. Estimates adjusted for area of residence, family structure, fathers' and mothers' education, religiosity and personality and fathers' heavy drinking occasions. Lifetime abstainers excluded. The results are available in tabular form from the corresponding author upon request.

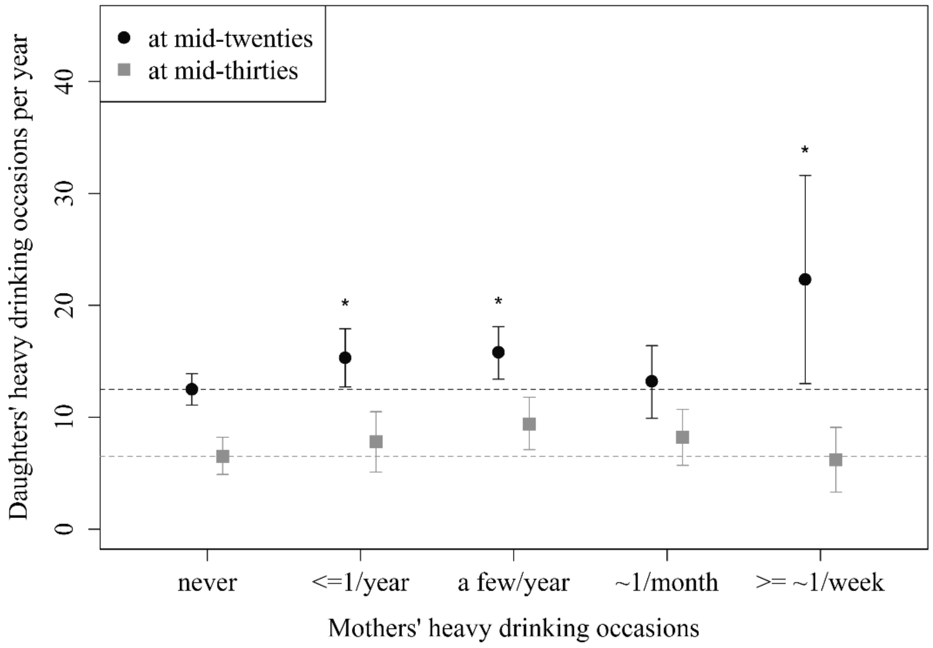

Supplement: Supplementary Material [file NIHMS1979828-supplement-Supplementary_Material.pdf]
